# APE1 controls DICER1 expression in NSCLC through miR-33a and miR-130b

**DOI:** 10.1007/s00018-022-04443-7

**Published:** 2022-07-25

**Authors:** Giulia Antoniali, Emiliano Dalla, Giovanna Mangiapane, Xiaolong Zhao, Xinming Jing, Yi Cheng, Veronica De Sanctis, Dilara Ayyildiz, Silvano Piazza, Mengxia Li, Gianluca Tell

**Affiliations:** 1 Laboratory of Molecular Biology and DNA Repair, Department of Medicine, University of Udine, Udine, Italy; 2 Cancer Center of Daping Hospital, Third Military Medical University, Chongqing, China; 3 Next Generation Sequence Facility, Department CIBIO, University of Trento, Trento, Italy; 4 Bioinformatics Core Facility, Department CIBIO, University of Trento, Trento, Italy; 5 Computational Biology, International Centre for Genetic Engineering and Biotechnology, ICGEB, Trieste, Italy

**Keywords:** miRnome, Cancer, Gene signatures, Network analysis

## Abstract

Increasing evidence suggests different, not completely understood roles of microRNA biogenesis in the development and progression of lung cancer. The overexpression of the DNA repair protein apurinic/apyrimidinic endodeoxyribonuclease 1 (APE1) is an important cause of poor chemotherapeutic response in lung cancer and its involvement in onco-miRNAs biogenesis has been recently described. Whether APE1 regulates miRNAs acting as prognostic biomarkers of lung cancer has not been investigated, yet. In this study, we analyzed miRNAs differential expression upon APE1 depletion in the A549 lung cancer cell line using high-throughput methods. We defined a signature of 13 miRNAs that strongly correlate with APE1 expression in human lung cancer: miR-1246, miR-4488, miR-24, miR-183, miR-660, miR-130b, miR-543, miR-200c, miR-376c, miR-218, miR-146a, miR-92b and miR-33a. Functional enrichment analysis of this signature revealed its biological relevance in cancer cell proliferation and survival. We validated DICER1 as a direct functional target of the APE1-regulated miRNA-33a-5p and miR-130b-3p. Importantly, IHC analyses of different human tumors confirmed a negative correlation existing between APE1 and Dicer1 protein levels. DICER1 downregulation represents a prognostic marker of cancer development but the mechanisms at the basis of this phenomenon are still completely unknown. Our findings, suggesting that APE1 modulates DICER1 expression via miR-33a and miR-130b, reveal new mechanistic insights on DICER1 regulation, which are of relevance in lung cancer chemoresistance and cancer invasiveness.

**Supplementary Information:**

The online version contains supplementary material available at 10.1007/s00018-022-04443-7.

## Introduction

Lung cancer is the most frequently diagnosed cancer and, in the past decades, the incidence and mortality of lung cancer have consistently increased worldwide. Non-small cell lung carcinoma (NSCLC) accounts for approximately 85% of all lung cancer occurrences [1]. Despite many achievements made in anti-cancer therapy over the years, the survival of NSCLC is still far from being satisfactory, due to the lack of effective prognostic and diagnostic tools. Currently, surgical resection remains the most effective treatment for early-stage NSCLC; however, many patients with NSCLC still develop tumor metastasis and recurrence after the pulmonary resection procedure [2]. Hence, exploring novel cancer-specific biomarkers for NSCLC patients would help monitor early diagnosis, tumor progression and guide tailored clinical treatments [2].

miRNAs are a class of small non-coding RNAs that participate in gene expression at the post-transcriptional level, by pairing to the 3′-untranslated region (3′UTR) of protein-coding mRNAs, thus causing their degradation or inhibiting protein translation [3]. Therefore, miRNAs play pivotal roles in a wide spectrum of biological processes including: cell proliferation and development, tumorigenesis, metastasis, invasion, and apoptosis [4–6]. For all these reasons, miRNAs have attracted great attention for their potentiality as novel biomarkers for diagnostic, prognostic, and therapeutic applications in multiple malignancies [7]. Recently, the application of high-throughput miRNA profiling methods has enhanced the identification of aberrantly expressed miRNAs in NSCLC [8–10] and the definition of several miRNAs as potential biomarkers for lung cancer [11, 12].

The cornerstone treatment for advanced NSCLC remains the platinum-based chemotherapy regimen, which relies on disrupting replication and transcription via intra- and interstrand DNA/RNA crosslinking, finally leading to cell death. However, there is a limited efficacy for platinum-based therapy due to inherited or acquired resistance. Several key factors contribute to chemoresistance; in particular, defects in cell cycle checkpoints and elevated DNA repair capacity are at the basis of platinum-based resistance [13]. For example, the expression and the activity of different base excision repair (BER) enzymes have been associated with NSCLC development and acquired resistance against chemotherapy and radiotherapy [14].

The human apurinic/apyrimidinic endodeoxyribonuclease 1 (APE1) is responsible for the protection of cells against genotoxins and for safeguarding genome stability as the main AP-endodeoxyribonuclease of the BER pathway [15] playing a pivotal role in cancer chemoresistance. Its overexpression has been identified in several cancer types including NSCLC [16, 17] and, in all cases, it is associated with a worse prognosis. Interestingly, APE1 levels have been reported to be a predictive marker for sensitivity to chemotherapy in NSCLC patients [18, 19]. More importantly, some studies have also reported that overexpression of APE1 is associated with increased EGFR-TKI-resistant cells due to epithelial-to-mesenchymal transition (EMT) mechanisms [20–22]. Since numerous evidence now recognizes that EMT not only contributes to metastasis but also to drug resistance processes, understanding the role of APE1 in lung cancer development is mandatory to use it as a promising therapeutic target for treating lung cancer patients.

Over the years, knowledge of APE1 biological functions, mechanisms of action, interactions and regulation has increased tremendously [23]. APE1 contributes to the regulation of oxidative stress responses and has other non-repair activities, such as direct and indirect modulation of the expression of chemo-resistance genes [24]. Recently, we and others have provided several lines of evidence suggesting that APE1 may modulate tumor progression and chemoresistance by controlling gene expression via unanticipated functions in RNA metabolism, including RNA processing for miRNA expression [25, 26]. In particular, we demonstrated that APE1’s endonuclease activity on the pri-miR-221/222 influenced the expression of the tumor suppressor phosphatase and tensin homolog (PTEN), thus impacting cell transformation [25]. Whether APE1 regulates miRNAs acting as a prognostic biomarker of lung cancer has not been investigated, yet.

In the present study, through an unbiased high-throughput miRNome profiling approach performed on NSCLC cells depleted of APE1 protein, we identified 13 putative miRNAs regulated by APE1. Then, we used miRNA–gene interaction networks, survival analysis based on TCGA datasets and pathway enrichment analysis to identify the putative gene targets of these miRNAs and to investigate their clinical-related aspects and their biological functions. In particular, we demonstrated that by regulating miR-33a and miR-130b expression levels, APE1 modulates DICER1 expression in cancer cell lines. Analysis of clinical cancer samples, which showed a direct correlation existing between APE1, miR33a, and miR-130b but an inverse correlation with DICER, supports a possible role for this axis in contributing to the acquisition of a malignant phenotype by lung cancer cells.

## Methods

### Cell lines and materials

A549 and CH12F3 cells were grown in RPMI medium (Euroclone, Milan, Italy), HeLa clones in Dulbecco's modified Eagle's medium (Invitrogen, Monza, Italy) while JHH-6 were cultured in William’s medium E (Sigma-Aldrich, St. Louis, MO). CH12F3 containing two (+ / + /Δ) and zero copies of APE1 (Δ/Δ/Δ) have been described previously [27]. All cells were supplemented with 10% fetal bovine serum (Euroclone), 1% penicillin–streptomycin solution (100 U/mL penicillin, 100 mg/mL streptomycin), 2 mM L-glutamine (Euroclone) and cultured in a humidified incubator at 5% CO_2_ at 37 °C. Cells were tested as free of mycoplasma contamination (N-GARDE Mycoplasma PCR Reagent, Euroclone).

For APE1 endonuclease activity inhibition, A549 cells were treated with 20 µM APE1 endonuclease inhibitor #3 [28] while 100 µM of E3330 [29] was used for redox activity inhibition.

### Transient transfections with siRNA, plasmids and miRNA mimic

One day before silencing, cells were seeded in 10-cm plates at a density of 3 × 10^6^ cells per plate. Cells were then transiently transfected with 100 pmol siRNA APE1 5ʹ-UACUCCAGUCGUACCAGACCU-3ʹ or the scramble control siRNA 5ʹ-CCAUGAGGUCAUGGUCUGdTdT-3ʹ (Dharmacon, Lafayette, CO) using DharmaFECT reagent (Dharmacon). After 72 h upon transfection, cells were collected and RNA extracted using miRNeasy kit (Qiagen, USA).

For the overexpression of the APE1 protein, A549 cells were transiently transfected with APE1 FLAG-tagged plasmid using the Lipofectamine 3000 reagent (Invitrogen), according to the manufacturer’s instructions and collected 24 h after transfection.

30 nM mimic hsa-miR-33a-5p, mimic hsa-miR-130b-3p or mimic negative control (Ambion) was transfected into A549 cells using Lipofectamine RNAi max (Invitrogen). Cells were incubated at 37 °C, 5% CO_2_ for 24 h.

### Cell viability and proliferation assay

Cell viability was measured using the 3 (4 5 dimethylthiazol 2 yl) 5 (3-carboxymethoxyphenyl) 2 (4-sulfophenyl) 2*H*-tetrazolium salt (MTS) assay (Celltiter 96 Aqueous One solution cell proliferation assay, Promega) on cells grown in 96-well plates. In detail, 5000 cells were plated on 96-wells and were allowed to attach to the plate for 24 h. The day after, cells were treated with either vehicle DMSO or increasing concentrations of APE1 inhibitors for 24 h. After treatment, the MTS solution was added to each well and the plates were incubated for 2 h at 37 °C. Absorbance was measured at 490 nm using a multiwell plate reader. All experiments were run in triplicates. The values were standardized to wells containing media alone and the cell viability was expressed as a fold change compared to the DMSO-treated cells.

### Determination of AP sites

Total abasic damage in chromosomal DNA was measured with an aldehyde-reactive probe (ARP). A549 cells were plated on 6-well plates and 24 h later were exposed to either vehicle DMSO or APE1 endonuclease inhibitor compound #3. Genomic DNA was isolated from A549 using QIAamp DNA Mini Kit (Qiagen) and then concentration and purity were determined by Nanodrop (Thermo Fisher Scientific). Samples of genomic DNA were analyzed using the DNA Damage Quantification Kit based on ARP (Dojndo, Gaithersburg, MD, USA), according to the manufacturer’s instructions. Briefly, 1 µg of genomic DNA was labeled with a biotinylated ARP for 1 h at 37 °C, and ARP-DNA was purified following the manufacturer’s instructions. The amount of labeled ARP-DNA was then quantified through a colorimetric reaction. Quantification of AP sites/cell was then measured using a calibration curve provided with the kit.

### Preparation of cell extracts and Western blotting analysis

Cell extracts were prepared and quantified as already described in [26]. For the preparation of whole cell lysate, the cell pellet was resuspended in lysis buffer containing 50 mM Tris–HCl (pH 7.4), 150 mM NaCl, 1 mM EDTA, 1% w/v Triton X-100 supplemented with 1 mM protease inhibitor cocktail (Sigma-Aldrich), 1 mM DTT, 0.5 mM phenylmethylsulfonyl fluoride (PMSF), 1 mM NaF and 1 mM Na_3_VO_4_ for 30 min at 4 °C. After centrifugation at 13,000 rpm for 20 min at 4 °C, the supernatant was collected as a whole cell lysate. The protein concentration was determined using Bio-Rad protein assay reagent (Bio-Rad, Hercules, CA, USA). The indicated amounts of whole cell extracts were resolved in 12% or 8% SDS-PAGE and transferred to nitrocellulose membranes (Sigma–Aldrich). Normalization was performed using either monoclonal anti-tubulin antibody (Sigma-Aldrich) or polyclonal anti-actin antibody (Sigma-Aldrich). Detection and quantification were performed with the Odyssey CLx Infrared imaging system (LI-COR GmbH, Germany) using Odyssey software (Image Studio 5.0). A list of the antibodies used is given in the Supplementary Information (Table S5). Original uncropped images of Western blots used in this study can be found in Supplementary Figures S9 and S10.

### RNA-seq

RNA-seq was performed in quadruplicate starting from 180 ng of total RNA from A549 cells silenced for the APE1 protein expression and from scramble transfected negative control. Purified RNAs were quantified with the Qubit RNA HS assay kit (Thermo Fisher Scientific). Sequencing libraries were prepared based on the SMARTer smRNA-Seq kit (Clontech/Takara, USA) protocol with minor changes enhancing the identification of pri-miRs in addition to miRNAs. The SMARTer smRNA-Seq kit utilizes a ligation-free ‘tailing approach’. First, the 3ʹ end is polyadenylated; subsequently, a reverse transcription (RT) reaction, primed by an oligo dT primer, incorporates the 3ʹ adapter. A specialized reverse transcriptase enzyme switches template upon reaching the end of each RNA template and utilizes the SMARTer smRNA-Seq oligo as a secondary template to attach the 5ʹ adapter. The size profiles of the individual libraries were analyzed with LabChip GX II using a DNA High Sensitivity kit (both PerkinElmer, USA). Libraries were quantified on a Qubit with the DNA High Sensitivity kit (Life Technologies).

Quantified libraries were mixed at an equimolar ratio and sequenced on the HiSeq 2500 (Illumina, USA) in rapid run mode, using a 100-bp, dual-indexed, single-end sequencing configuration.

The FastQC tool (https://www.bioinformatics.babraham.ac.uk/projects/fastqc/, version 0.11.6) was used to evaluate fastq files quality and the output was summarized with multiQC (http://multiqc.info/, ver1.4). Reads had very good quality and no correction was required. We used Cutadapter (https://cutadapt.readthedocs.io, ver 1.15) to remove adapters, primers, poly-A tails, and other types of unwanted sequences from the fastq files. Transcript quantification was conducted with STAR (v2.5.3a) [30], using the human genome assembly GRCh38 with reference annotation; reads were assigned to a gene-based on EnsEMBL annotation and via the STAR function “quantMode GeneCounts”. Differential expression (DE) analysis was performed using gene raw counts, within the R/Bioconductor DESeq2 package [31]: we estimated the dispersion parameter for each library using the biological group dispersion; abs(log2(fold change)) ≥ 0.75 was considered for differentially regulated genes; we adjusted the P value for multiple testing using the Benjamini–Hochberg correction with a false discovery rate (FDR) ≤ 0.05.

### NanoString nCounter system miRNA Assay

miRNA expression profiling was performed with 100 ng of total RNA from A549 cells silenced for the APE1 protein expression and from scramble transfected negative control. The experiment was performed in triplicates. RNA was isolated using the miRNeasy kit (Qiagen, USA) and samples were prepared for the nCounter miRNA expression profiling using the human v3 miRNA expression panel, according to the manufacturer’s recommendations (NanoString, Seattle, Washington, USA) in the SynLab Srl. Transcript counts were normalized through the normalization method incorporated in the model framework, estimating parameters from positive controls, negative controls, and housekeeping genes embedded in the nCounter system, using the NanoStringDiff package [32] within Bioconductor. Differential expression of genes was assessed on log2-normalized data with a generalized linear model likelihood ratio test, using the glm.LRT function within the NanoStringDiff package. A q-value cutoff of 0.1 was used to determine statistical significance. For clustering analysis, we used the normalized values generated by the NanoStringDiff package. Starting from the log2-normalized values, genes with low standard deviation (SD < 0.2) were filtered out and hierarchical clustering of the samples was performed and visualized as a heatmap of log2-normalized, centered, and scaled in the row direction values using the heatmap.2 function within the gplots R/Bioconductor package (Euclidean distance, Complete linkage) [33]. Principal components analysis was also performed to evaluate biological replicates’ reproducibility. Independent validation analysis on 13 differential miRNAs was performed through qRT-PCR.

### Construction of DE-miRNAs and APE1 PPI targets network

To determine the relationship between APE1-interacting protein communities and DE-miRNAs in both NanoString and RNA-seq experiments, we constructed a miRNA-PPI network for the LUAD dataset. For this purpose, experimentally retrieved APE1-interacting partners were initially used to establish the global APE1 protein–protein interaction network using the InWeb_InBioMap [34] and Cytoscape (v3.6.1) tools [35] as described in detail in [36]. Briefly, the differential gene expression results from TCGA and normal datasets (GTEx data) for the genes encoding the proteins present in the APE1-PPI network were obtained via the GDC data portal and the RUVSeq R/Bioconductor package [37] was used to eliminate the batch effect coming from the combination of two data sources. Kaplan–Meier curves were plotted for each differentially expressed gene by RTCGA R/Bioconductor package [38]. As a result, we selected genes significantly differentially expressed (*p* < 0.05, absolute log fold change > 1) and associated with a bad prognosis. The up-regulated and poor prognostic APE1-PPI were selected as poor prognostic markers. miRNAs targeting the genes of the constructed network were then retrieved by mirWalk v.3.0 [39] and DIANA-Tarbase v.8.0 tools following the authors’ recommendations [40]. DE-miRNAs identified in RNA-seq and NanoString experiments were selected from those previously retrieved and were used to build the final DE-miRNAs – APE PPI targets network (Nproteins = 96 and NmiRNAs = 42).

### RNA extraction and quantitative Reverse Transcriptase-PCR (qRT-PCR)

For miRNAs and RNAs qRT-PCR analysis from in vitro cultured cell lines, RNA was isolated using miRNeasy kit (Qiagen, USA), according to the manufacturer’s instructions.

Selected candidate miRNAs were validated by RT-qPCR using TaqMan Advanced miRNA assay (Life Technologies, Carlsbad, CA, USA) following the manufacturer's instructions. Detection of successfully transcribed products was carried out using TaqMan Fast Advanced Master Mix and CFX Touch™ Real-Time PCR System (Bio-Rad, Hercules, CA). qRT-PCR results were calculated using the ΔΔct method, utilizing the expression of miR-16-5p as reference.

For the measurement of mRNA expression, one microgram of total RNA was reverse transcribed using the SensiFAST cDNA synthesis kit (Bioline, London, UK), according to the manufacturer’s instructions. qRT-PCR was performed with a CFX96 Real-Time System (Bio-Rad) using SensiFAST SYBR No-ROX kit (Bioline, London, UK). The primers and probes used are listed in Supplementary Information (Table S6).

### MiRNA extraction and analysis from tissue samples

Total RNA was extracted from formalin-fixed paraffin-embedded (FFPE) samples using QIAGEN’s RNeasy FFPE kit (RNeasy FFPE, Hilden, Germany) following the manufacturer’s instructions. cDNA was synthesized from 200 ng total RNA and amplified by RT‑qPCR using TB Green PremixExTaq II (Takara Bio Inc., Japan). The thermocycling condition for miRNA consisted of 95˚C for 20 sec followed by 40 cycles of 10 sec at 95˚C, 20 sec at 60˚C and 10 sec at 70˚C. For normalization, U6 was used as an internal reference control. All the primers were designed by Biowavelet Ltd., Chongqing, China and synthesized by Tsingke Biotechnology Ltd., Beijing, China. The expression level of miRNAs was calculated using the log_2_(2^−ΔCt^ × 10^10^) formula based on the previous description [41].

### Cancer specimens and immunohistochemistry

One hundred paraffin-embedded cancerous tissue samples, including colorectal cancer, glioblastoma, breast cancer, cervical cancer, and NSCLC were collected from patients who underwent surgical resection without prior chemotherapy or radiotherapy in Daping Hospital, Third Military Medical University (Chongqing, China) from 2015 to 2016. This study was approved by the Ethics and Research Committee of the Daping faculty of Medicine, Third Military Medical University, Chongqing, China; written informed consent was obtained from all patients. The Histopathological assessment was carried out separately by two pathologists and then a consensus was made on discordant assessments. Sections from formalin-fixed and paraffin-embedded (FFPE) tumors were incubated with primary antibodies overnight, at 4 °C. Antibodies were purchased from Abcam (Cambridge, MA), unless indicated otherwise. All antibodies used for the immunohistochemistry are listed: APE1 antibody (clone 13B8E5C2; dilution 1:5000; Novus Biologicals), Dicer1 antibody (clone13D6; dilution 1:50; Abcam, Cambridge, MA), ADM antibody (10778-1-AP; dilution 1:100; Proteintech), CDKN1A antibody (ab109520; dilution 1:100; Abcam), CCN2 antibody (ab6992; dilution 1:100, Abcam), DICER1 antibody (ab259327; dilution 1:100, Abcam), FLT1 antibody (ab259327; dilution 1:250, Abcam), JAG1 antibody (ab7771; dilution 1:100, Abcam) and TGM2 antibody (ab2386; dilution 1:50, Abcam). Sections were rinsed with PBS and incubated with goat anti-mouse secondary antibody. Sections were rinsed with PBS, developed with diaminobenzidine substrate, and then counterstained with diluted Harris hematoxylin. APE1, DICER1, ADM, CDKN1A, CCN2, FLT1, JAG1, and TGM2 staining were analyzed and scored for four categories: (i) score 0, no expression in tumor cells; (ii) score 1 + , faint/barely perceptible partial expression in < 10% of tumor cells; (iii) score 2 + , weak to moderate expression in > 10% of tumor cells; (iv) score 3 + , strong expression in > 10% of tumor cells. Image analysis was done by two experienced pathologists independently.

### Survival analysis

The prognostic value of selected DE-miRNAs was first evaluated singularly in the TCGA-LUAD lung adenocarcinoma dataset using the YM500v3 database [42]. Using the “Survival” function, we split patients by median, upper- or lower-tertile expression levels, drawing Kaplan–Meier plots and assessing the statistical significance of each curve.

miRNA expression data (HiSeq, miRgene level; RPM, Log2(Val + 1), miRNA expression for tumor samples (Illumina HiSeq platform, miRgene level, Normalized, RPM), and clinical data were downloaded from the LinkedOmics portal for TCGA-LUAD patients (*n* = 450 and *n* = 522, respectively; http://linkedomics.org/data_download/TCGA-LUAD/; last accessed: January 24, 2022).

The prognostic value of the thirteen-candidate DE-miRNAs signature was evaluated using the RTCGA.clinical (providing clinical datasets from The Cancer Genome Atlas Project for all cohort types) and survival R packages (containing the core survival analysis routines, including definition of Surv objects, Kaplan–Meier and Aalen–Johansen (multi-state) curves, Cox models, and parametric accelerated failure time models). We first applied a Cox proportional hazard model and defined, for each miRNA, the multivariate analysis Cox coefficient; we then multiplied this coefficient by the expression value of the associated miRNA for every patient in the TCGA-LUAD dataset, obtaining the miRNA score; finally, the sum of all miRNA scores provided the Prognostic Index (PI) of each patient. We separated patients into “high-risk” and “low-risk” based on *p* value optimization using the ‘surv_cutpoint’ function (minprop = 0.33). The difference in overall survival rates between the two subgroups was verified by applying a log-rank test and a Kaplan–Meier plot was finally drawn to summarize the data.

### miRNA targets functional enrichment analysis

DE-miRNA human validated targets were retrieved through the DIANA-MirPath v.3 web server [43]. Functional enrichment analysis was performed by querying the KEGG and Gene Ontology – Biological Process databases (*p* ≤ 0.05), applying the “genes union” and “pathways union” methods. A reduced graphical representation of raw data was obtained using KEGG-PathwayConnector [44] (sorted by ascending Adjusted *p* value; number of EnrichR pathways to analyze: 10) and REVIGO [45] (default settings). Additional information on expressed DE-miRNA targets was retrieved from the Molecular Signatures Database v6.2 Hallmark, Computational, Oncogenic, Immunologic and Chemical and Genetic Perturbations (C4, C6, C7, CGP, H) collections (top 20 gene sets, FDR *q* value ≤ 0.05) [46, 47].

### Definition of the EMT consensus signature

We assembled an “EMT Consensus Signature” (*n* = 1407) through data mining and union of the following datasets: (a) genes associated with “Adherens Junctions” (https://www.genome.jp/dbget-bin/www_bget?hsa04520; *n* = 71) and “Focal Adhesion” (https://www.genome.jp/dbget-bin/www_bget?hsa04510; *n* = 199) in the KEGG database (last accessed: February 25, 2020); (b) genes associated with “epithelial-to-mesenchymal transition” (http://amigo.geneontology.org/amigo/term/GO:0001837; *n* = 181) in the Gene Ontology database (last accessed: February 26, 2020); (c) complete list of EMT-associated genes from dbEMT (http://dbemt.bioinfo-minzhao.org/index.html; *n* = 1185; last accessed: February 26, 2020) [48, 49].

### Definition of the 13 DE-miRNAs expressed validated targets EMT model network

The fifteen expressed validated targets of the 13 DE-miRNAs signature, which were included in the EMT signature, were used as input gene list of the InWeb_InBioMap [34]. Settings for network construction were as follow: database version 2020_03_04; Network Expansion to Include neighboring proteins; Relevance Score Type inclusive; Relevance score cutoff 0.8. Functional enrichment analysis was performed using the InWeb_InBioMap built-in tool, focusing on statistically significant, biologically relevant annotations obtained from all the queried databases.

### Gene expression profiling of EMT-related expressed validated targets in TCGA and GTEx datasets

Gene expression data of the fifteen EMT-related expressed validated targets were obtained, for the TCGA-LUAD dataset (*n* = 483) and for the matched TCGA normal and GTEx data (*n* = 347), querying the GEPIA2 web tool ([50], sequentially selecting “Expression Analysis, Expression DIY, Box Plots, Signatures” and finally copy-pasting the gene symbols of interest in the “Gene Set A” box. The output was represented as boxplots (red: tumor; black: normal).

### Statistical analysis

The results are presented as means ± S.D., and data analysis was performed with the Prism GraphPad 7.0 software. For comparisons between two groups, unpaired and paired Student’s *t*-tests were used. In all tests, *p* values < 0.05 were considered statistically significant. *p < 0.05; **p < 0.001.

## Results

### Analysis of miRNA expression profiles in A549 lung cancer cells upon APE1 knockdown

**Fig. 1:**
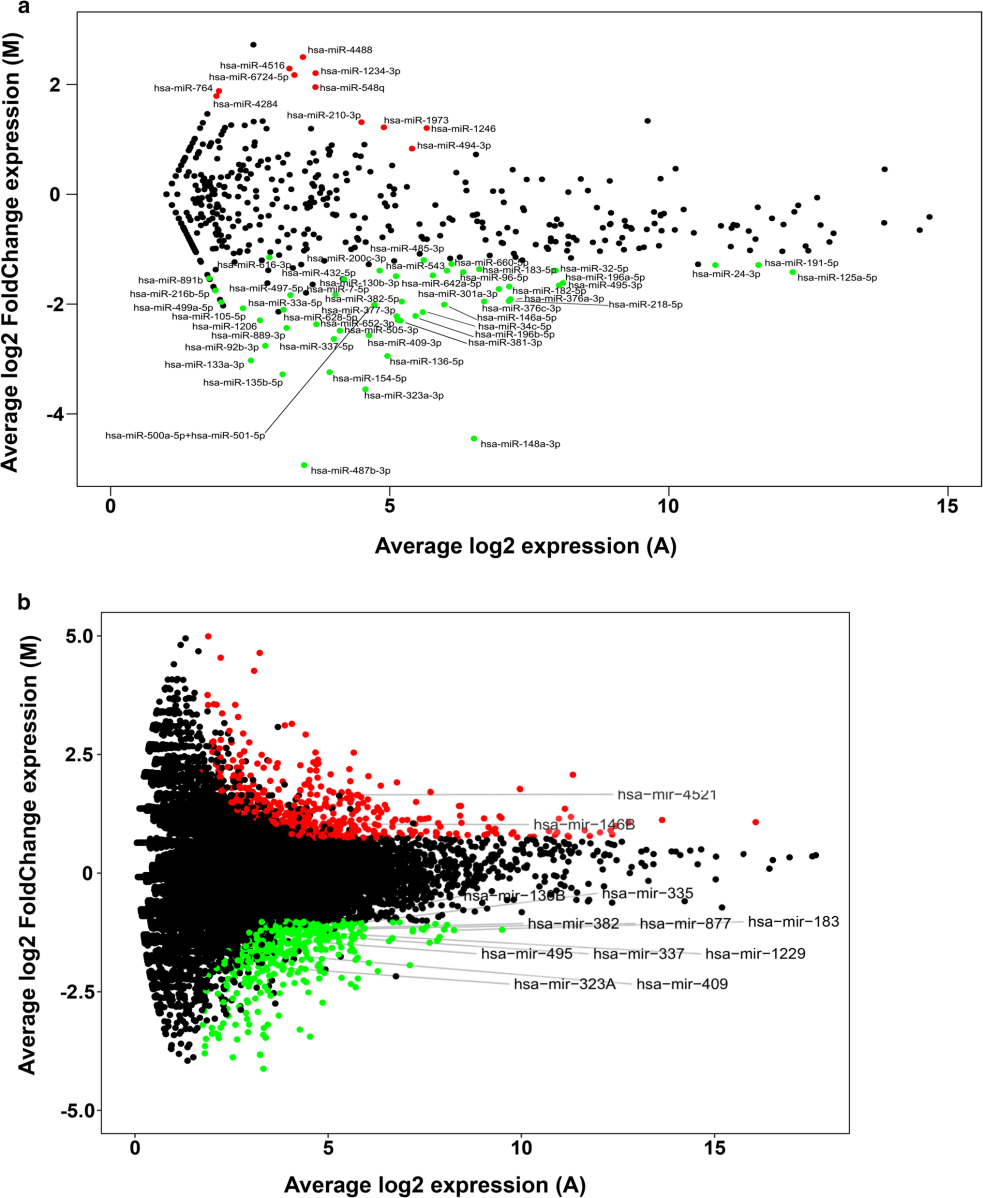
Global profiling of microRNA expression in A549 cells APE1-depleted. **a** MA plot showing the average fold change (log2 scale, *y*-axis) and the average expression (log2 scale, *x*-axis) of the 798 miRNAs profiled in the NanoString experiment. DE-miRNAs with multiple-test adjusted-pvalue less than 0.1 and log2 fold change greater or lower than 1 are indicated in red (up-regulated in siAPE1, *n* = 11) and green (down-regulated in siAPE1, *n* = 50), respectively. **b** MA plot showing the average fold change (log2 scale, *y*-axis) and the average expression (log2 scale, *x*-axis) of all the transcripts profiled in the RNA-seq experiment. RNA-seq features with multiple-test adjusted-pvalue less than 0.05 and log2 fold change greater or lower than ± 1.0 are indicated in red (up-regulated in siAPE1, *n* = 316) and green (down-regulated in siAPE1, *n* = 421), respectively. DE-miRNAs with at least 10 average counts in the samples are indicated by their corresponding labels

Previous studies showed that APE1 expression is up-regulated in different tumor tissues, including lung cancer [19]. More interestingly, recent works supported a role for APE1 in miRNAs processing involved in chemoresistance [25, 51]. However, the specific involvement of APE1 in the expression of miRNAs in NSCLC tissues and cell lines has not been elucidated, so far. In this present study, the A549 cell line was used to evaluate differentially expressed miRNAs (DE-miRNAs) upon APE1 depletion. A549 were transiently silenced for the expression of the APE1 protein (Fig. S1A) and both NanoString (abs(log2FC) ≥ 1.0, *q*-value ≤ 0.1) and RNA-seq (abs(log2FC) ≥ 1.0, *q*-value ≤ 0.05) analyses were performed to identify DE-miRNAs occurring between APE1-siRNA silenced and scramble-siRNA cells (Fig. 1; Table S1–2). By comparing both the hierarchical-clustering and the principal components analysis, we were able to confirm the good reproducibility of all biological replicas (Fig. S1B–C).

Among the 798 miRNAs profiled through the NanoString technology, a total of 61 miRNAs resulted differentially expressed in a statistically significant manner, including 11 up-regulated and 50 down-regulated miRNAs (Fig. 1A and Table S1). Moreover, the RNA-seq analysis identified 12 miRNAs that were significantly dysregulated (10 had decreased expression and 2 were up-regulated) (Fig. 1B and Table S2). By comparing the lists of DE-miRNAs obtained through the two methodologies, 11 out of 12 miRNAs found in the RNA-seq analysis were also confirmed by NanoString and showed the same trend of down-regulation, although only 7 were statistically significant (miR-337, miR-323A, miR-409, miR-382, miR-495, miR-130b and miR-183). miR-1229, found in RNA-seq analysis, was not profiled through NanoString.

Several publications have disclosed inconsistencies among the amounts of miRNAs present in the original samples and those identified using different analysis platforms [52], sequencing approaches [53, 54] and even library preparation protocols [55]. Most of these discrepancies are in the miRNA detection rate sensitivity and differential expression. Moreover, in our RNA-seq library preparation, the protocol was also modified to allow the detection not only of miRNAs but also pri-miRNAs and other kinds of RNAs; therefore, a complete overlap with the NanoString DE-miRNA outcomes was not expected.

### Identification of candidate prognostic miRNAs for NSCLC

To identify a signature of candidate miRNAs having a potential prognostic value, we evaluated different features (Table 1). First, we queried the YM500v3 database [42] to correlate miRNA expression data and survival, according to TCGA datasets. Nine DE-miRNAs (miR-1246, miR-4488, miR-660, miR-218, miR-543, miR-200c, miR-376c, miR-376a, and miR-146a) showed a significant correlation between poor survival and miRNA expression in the TCGA-LUAD lung adenocarcinoma dataset (Fig. S2). Second, we compared DE-miRNAs identified by NanoString in A549 cells with those previously found in APE1-depleted HeLa cells [25], to highlight putative common regulators of tumor progression, picking out 11 miRNAs: 10 showed the same trend of down-regulation (miR-24, miR-301a, miR-196b, miR-500a + miR-501, miR-505, miR-628, miR-92b, miR-33a, miR-660, miR218), while only miR-1246 showed an opposite trend. Three of these miRNAs (miR-1246, miR-660-5p, and miR-218-5p) were also included among those having TCGA-prognostic values. Finally, we reviewed existing literature demonstrating an involvement of these miRNAs in chemoresistance processes [56–59]. Based on all these assumptions, we finally selected a group of 13 DE-miRNAs for further studies (Table 1). Interestingly, many of them were already reported in the literature to be altered in several cancer types (Table 1), implying the possible existence of common regulators/pathways involved in multiple malignancies and leading to the onset of drug resistance mechanisms through APE1 regulation.

**Table 1:**
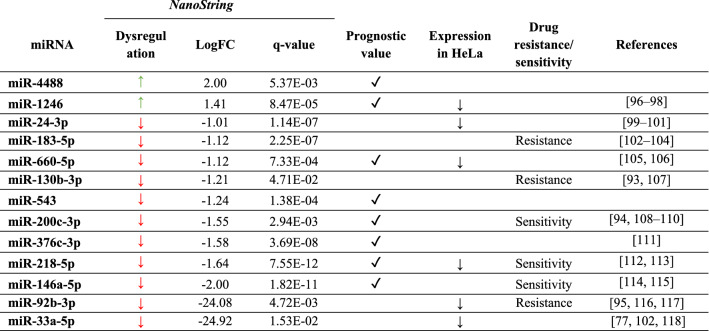
List of candidate miRNAs selected

**Fig. 2:**
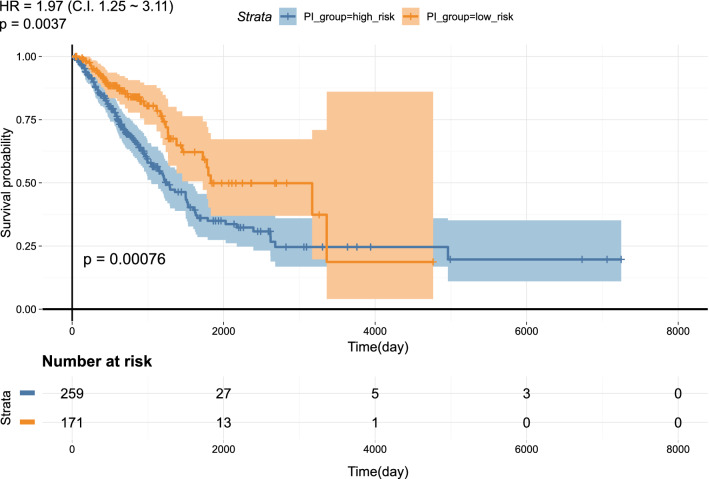
Prognostic value of the miRNA signature in TGCA-LUAD patients. Kaplan–Meier plot showing the different overall survival rates of patients belonging to the “high risk” and “low risk” groups, stratified based on the Prognostic Index calculated from the thirteen-candidate miRNA signature (see Fig. S2)

To further explore the prognostic power of candidate DE-miRNAs in TCGA-LUAD patients, we developed a prognostic index (PI) to associate patients’ overall survival to the DE-miRNAs signature (miR-4488 was excluded from the analysis since no data were available). In particular, we calculated patients’ PI using Cox regression coefficients and the expression values of DE-miRNAs. The risk groups were defined by stratifying patients based on *p* value optimization and a Kaplan–Meier plot was generated, with overall survival rates that were clearly different between high-risk (*n* = 259) and low-risk (*n* = 171) patients (*p* value = 0.00076) (Fig. 2, Fig. S2 and Table S3).

These findings support the potential prognostic value of the DE-miRNAs signature correlated with APE1 expression.

### Confirmation of DE-miRNAs status and clinical outcome

**Fig. 3:**
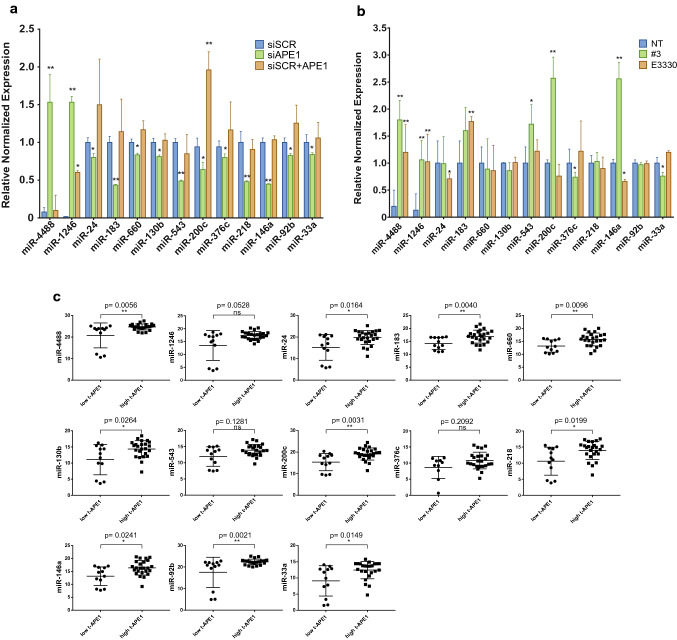
Validation of APE1 functional involvement in selected DE-miRNA expression. **a** RT-qPCR analysis on the thirteen selected DE-miRNAs was performed on A549 cells silenced for APE1 (siAPE1) or transiently transfected with the expression plasmid for the FLAG-tagged siRNA-resistant APE1 protein APE1^WT^ (siSCR + APE). Histograms report data using the ΔΔCT method with miR-16-5p as the reference. A two-sample, two-tailed, unpaired t test was used to compare the ΔΔCT values from each group with respect to cells transfected with scramble siRNA (siSCR). **b** RT-qPCR analysis on the thirteen selected DE-miRNAs performed on A549 cells treated with 20 µM #3 and 100 µM E3330 for 24 h, respectively (see also Fig. S3B-C). **c** Correlative expression of APE1 and DE-miRNAs in a cohort of human NSCLC specimens. Scatter plots report for each DE-miRNA the expression levels detected in the two cohorts of low- and high-APE1 protein (see also Fig. S4). Statistical significance is represented as *p < 0.05, **p < 0.01

Based on previous results, we first experimentally verified the expression levels of these 13 candidate miRNAs in the RNA samples used for high-throughput analysis, by qRT-PCR, using miRNA-specific TaqMan probes in pooled samples (Fig. S3A). Consistently, qRT-PCR results were in accordance with the RNA seq and NanoString expression levels for all the 13 miRNAs tested. Simultaneously, data were also validated in an independent experimental data set, in which we both downregulated, through specific a siRNA, or overexpressed the APE1 protein through the use of a specific FLAG-APE1 expressing plasmid (Fig. 3A). As expected, APE1 silencing confirmed the transcriptomic results, while the overexpression of APE1 resulted in an increased expression of miRNAs that were down-regulated upon APE1 silencing, supporting the hypothesis that the expression of the selected miRNAs indeed depends on APE1 extent.

To better define the role of APE1 in processing DE-miRNAs, we tested whether its endonuclease or redox activities were involved. For this purpose, A549 cells were treated with two APE1 inhibitors: (i) Compound #3, a catalytic inhibitor of APE1 endonuclease activity [28] and (ii) E3330, a well-known inhibitor of APE1 redox activity now used in clinical trials [29]. Cells were challenged with APE1 inhibitors for 24 h and the thirteen selected DE-miRNAs were quantified through qRT-PCR (Fig. 3B). Time and doses of treatments were chosen based on their effect on cell viability and previously published data [25]. The efficacy of the treatments was evaluated by measuring the accumulation of AP sites generation, in the case of Compound #3, and the expression levels of Survivin, a known target of the APE1 redox function, in the case of E3330 [25, 60] (Fig. S3B, C). Treatment with Compound #3 resulted in an increased expression of miR-1246 and miR-4888; this was also apparent when A549 cells were challenged with APE1 redox inhibitor E3330, making it difficult to delineate which of the two APE1 functions could be involved in the expression of these miRNAs. For the other selected DE-miRNAs, few significant differences were observed with respect to non-treated cells: miR-33a and miR-376c were down-regulated when APE1 endonuclease function was inhibited, while miR-24 and miR-146a seemed to be regulated by the redox function of the protein. Two opposite results were observed for miR-200c and miR-146a, in comparison to those obtained upon APE1 depletion. Further experiments are required to better circumstantiate these results.

To further investigate the oncogenic relevance of the thirteen DE-miRNAs identified in this work, we evaluated their expression along with APE1 protein levels in a large cohort of NSCLC patients' tissues. Tissue samples were obtained from surgical resection specimens of NSCLC. According to the results of the immunohistochemistry (IHC) analysis, patients were divided into two groups: the low tissue APE1-expressing group (low-t APE1) and the high tissue APE1-expressing group (high-t APE1) (Fig. S4A). Firstly, we defined the IHC score of 0–1 as low-t APE1 group (*n* = 12), and 2–3 as high-t APE1 group (*n* = 24). The results showed the existence of 10 miRNAs (miR-24, miR-33a, miR-92b, miR-130b, miR-200c, miR-146a, miR-660, miR-218, miR-4488, and miR-183) that were expressed at significantly higher levels in the high-t APE1 group than in the low-t one (Fig. 3C). For three miRNAs (miR-1246, miR-376c, and miR-543), a non-significant difference was found between the two cohorts, while for miR-4488 an opposite trend was observed. Considering the large number of high-t APE1 patients, we defined score 3 as the high-t APE1 group (*n* = 11). Similar significant results were obtained for 10 miRNAs (Fig. S4B). Altogether, these results suggest that the expression levels of the large majority (10 out of 13) of tissue miRNAs and APE1 are related, providing clinical relevance to our in vitro data.

Thoroughly, these results mirror an oncogenic potential role for candidate APE1-regulated-miRNAs in lung cancer.

### APE1 cellular depletion affects the expression of genes related to miRNA processing

**Fig. 4:**
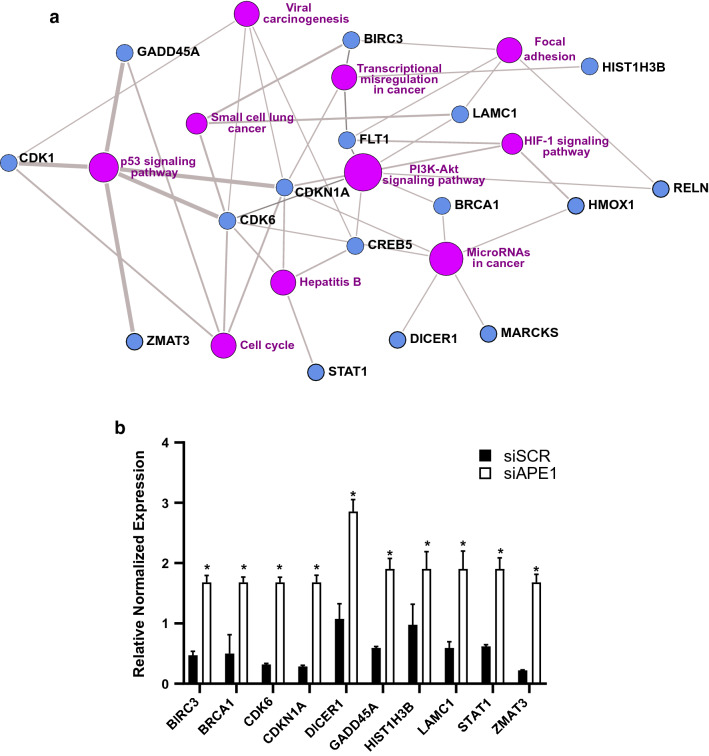
Functional enrichment analysis of DE-miRNAs expressed validated target. **a.** Network of the top10 enriched KEGG functional terms (*p* value ≤ 0.05) associated with the expressed validated targets (*n* = 74) of DE-miRNA, according to KEGG-PathwayConnector. Ten major clusters (purple nodes) can be defined including tumorigenic pathways (e.g., p53, Hippo, HIF1A, and PI3K-Akt signaling), metabolic and structural events associated with the cell cycle and immune response. The role of miRNAs in non-small cell lung carcinoma is also identified. In addition, the genes associated with each enriched functional term are reported (blue nodes). **b** Gene expression profiling of APE1 DE-miRNAs targets in A549 cells. A549 cell line was silenced using APE1 siRNA for 72 h and mRNA expression levels were assessed by Real-Time qPCR and normalized with GAPDH. *p < 0.01

To investigate the role of DE-miRNAs in cellular processes, we assessed the enriched biological functions associated with their target genes by focusing on those involved in lung tumor progression and chemoresistance. For each DE-miRNA, we defined its validated targets and common enriched functional terms, as described in the Methods section. Interestingly, along with the identification of tumorigenic pathways (e.g., p53 and Hippo signaling) and metabolic/structural events associated with the cell cycle (Table S4), we also found immune-related terms implying the existence of a complex immune scenery in NSCLC, possibly having major implications in setting up protocols for immune-based precision medicine strategies [61]. We then specifically focused on the subset of targets (*n* = 74) differentially expressed in the A549 cell line based on RNA-seq results (abs(logFC) ≥ 1.0, *q* value ≤ 0.05). In particular, considering all the differentially expressed genes (DEGs), we only retained those having an anti-correlated expression compared to their regulatory miRNAs. We confirmed the previous results (Fig. 4A and Table S4), likely identifying some real effectors of those phenotypes (e.g., CDK1, CDK6, and CDKN1A): for each functional node (violet spheres), representing a dysregulated pathway, the network also shows some of the major target genes that were affected (blue spheres). Consistently, qRT-PCR analysis confirmed the upregulation of DE-miRNA targets when cellular APE1 was depleted (Fig. 4B). Thus, the overall characterization of tumor-related pathways was improved, now including also the HIF1A and the PI3K-Akt pathways in the pool of those affected. We also obtained clear evidence for the association of expressed target genes with miRNAs involved in cancer and small cell lung cancer, thus emphasizing the role of the deregulated miRNAs/mRNAs axis in the development and maintenance of lung cancer (Fig. 4A and Table S4). Finally, two other important nodes were represented by focal adhesion and miRNA processing, this is in accordance with several works pointing to a role of APE1 in the epithelial–mesenchymal transition (EMT) process [20, 22, 62, 63] and in APE1 contribution to miRNA biogenesis [25].

### APE1-mediated miRNA regulation is associated with the promotion of an EMT program

**Fig. 5:**
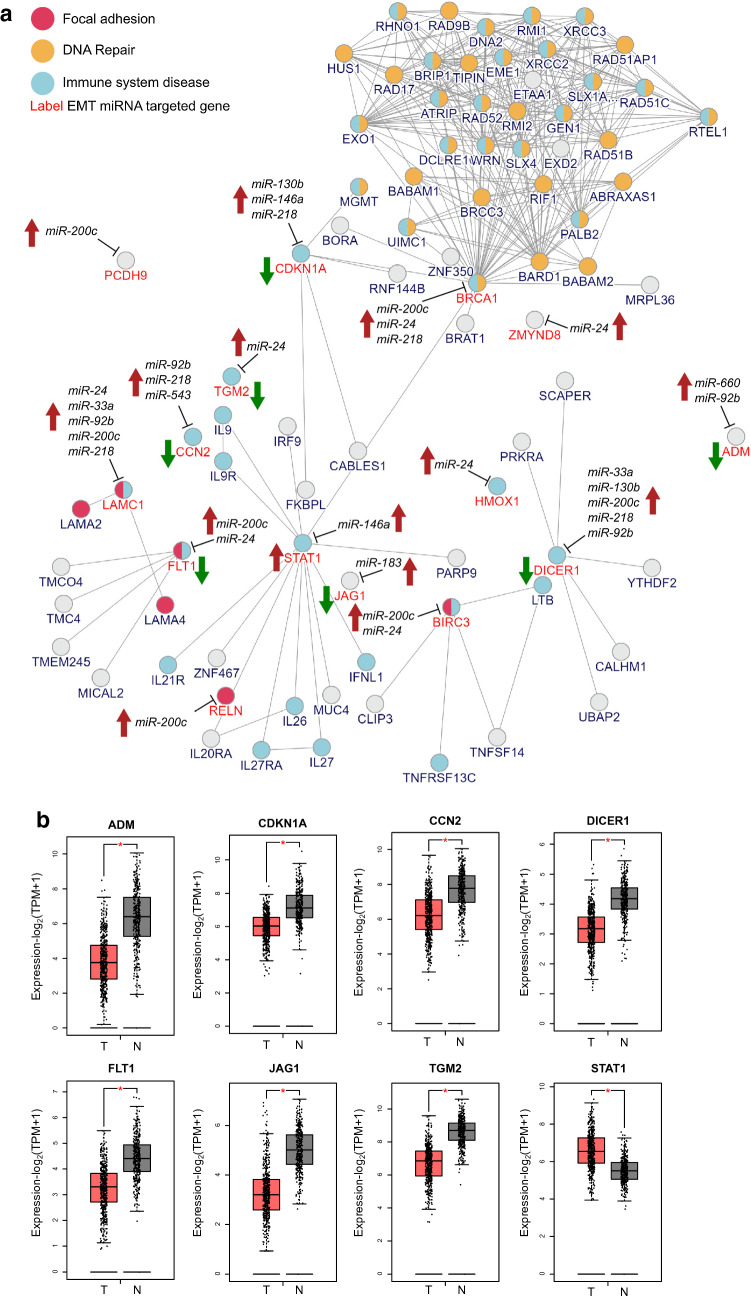
Role of APE1 in the regulation of Epithelial–Mesenchymal Transition. **a** Network showing the interconnections between APE1-regulated miRNAs (*n* = 10), their expressed validated target genes involved in EMT (*n* = 15, red label) and additional interactors (black label), according to the InWeb_InBioMap tool. Arrows close to miRNAs indicate the effect of APE1 regulation on miRNA expression (red: up-regulation); arrows close to target genes indicate the statistically significant expression status in TCGA-LUAD samples compared to matched TCGA and GTEx normal data (red: up-regulation; green: down-regulation). Nodes were functionally annotated using the InWeb_InBioMap built-in tool and genes associated with immune response and DNA repair are shown in turquoise and orange, respectively (*p* value < 0.05) **b** Gene expression profiling of EMT-related expressed validated targets in the TCGA-LUAD dataset. Boxplots showing the log2-transformed gene expression levels of the eight EMT-related expressed validated targets having significant differences in the TCGA-LUAD (*n* = 483) compared to the matched TCGA normal and GTEx datasets (*n* = 347). Red: tumor; black: normal. *p < 0.01

Previous studies highlighted the APE1 association with increased EGFR-TKI-resistant cells due to epithelial-to-mesenchymal transition mechanisms [20, 21]. In agreement, we demonstrated that, in our cellular models, APE1 depletion was associated with the upregulation of the epithelial marker E-cadherin, while its overexpression resulted in the upregulation of the mesenchymal marker Vimentin, which also correlated with the increased expression of three EMT-related transcription factors ZEB1, ZEB and SNAL1 (Fig. S5). Considering the importance of EMT as a crucial process for drug resistance, and that several miRNAs co-regulate both EMT and chemoresistance processes, we assembled an “EMT Consensus Signature” through data mining of several public databases. We then evaluated how many expressed and validated targets of the thirteen DE-miRNAs were included in the EMT signature. We found fifteen hits and used them for building a network model of the EMT pathway, thus recapitulating: (i) APE1-regulated miRNAs, (ii) their validated target genes and iii) additional interactors, putatively involved in the same biological processes (Fig. 5A). Finally, we annotated the network nodes, in search of additional relevant pathways significantly enriched. Interestingly, DNA repair and the immune response were two other functions associated with many nodes, suggesting that APE1 regulation could also affect the tumor microenvironment at different levels. DICER1 and LAMC1 were the nodes regulated by the highest number of DE-miRNAs, including miR-130b and miR-33a, followed by CCN2 and CDKN1A; STAT1 represented the central node of the EMT/immune subnetwork, connected through BRCA1 to immune/DNA repair nodes.

Notably, we also evaluated the expression levels of validated target genes in the TCGA-LUAD dataset, compared to matched TCGA and GTEx normal data (Fig. 5B), and found that seven genes (ADM, CDKN1A, CCN2, DICER1, FLT1, JAG1, and TGM2) were significantly down-regulated in the tumor, while STAT1 was the only up-regulated one. These results confirmed what we previously observed in A549 cells, indicating that this network module highlights a likely contribution of APE1 to the regulation of miRNAs function in lung cancer progression.

**Fig. 6:**
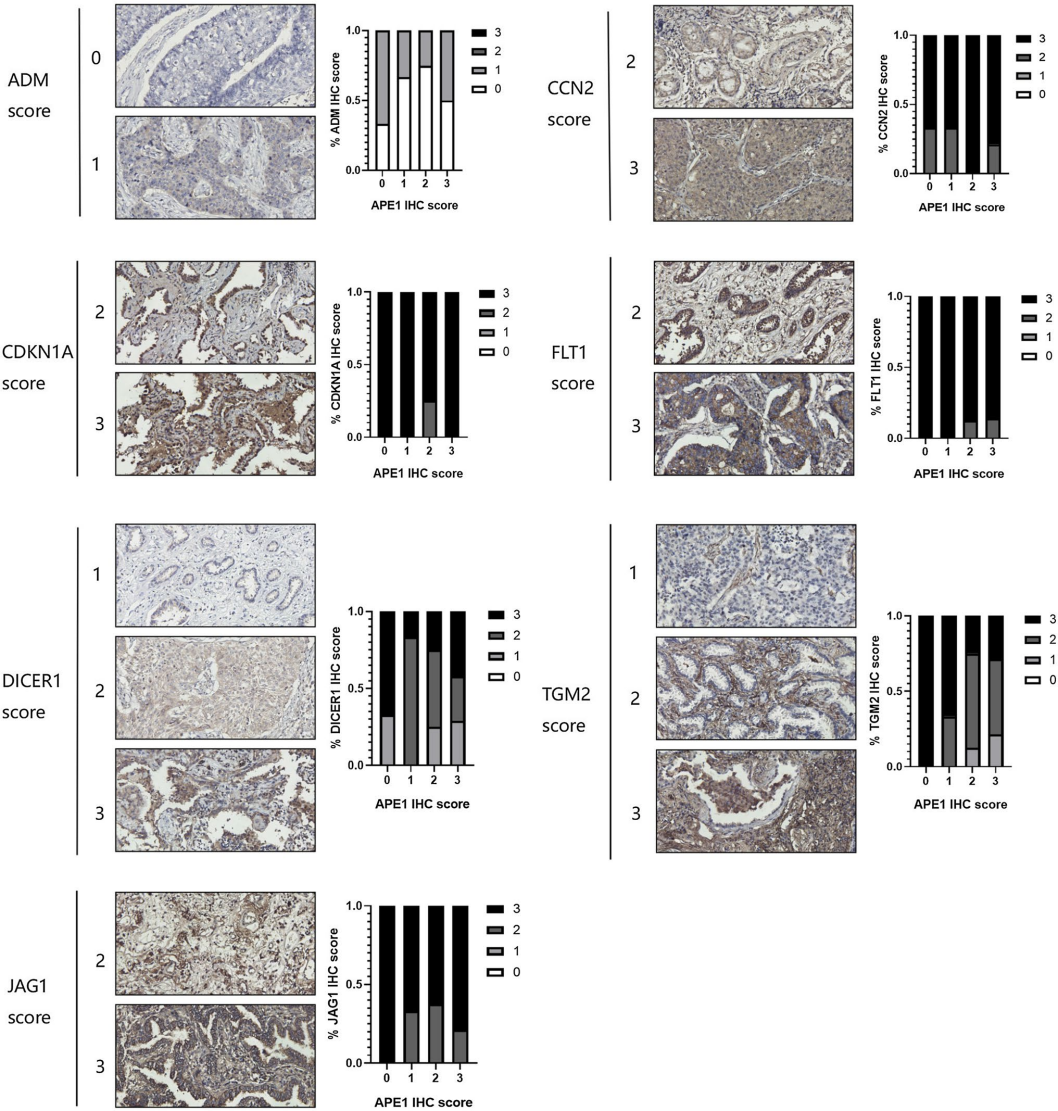
Correlative expression of APE1 and EMT genes. Representative images of APE1-high and -low examples for ADM, CDKN1A, CCN2, DICER1, FLT1, JAG1, and TGM2 protein expression determined by IHC assay were shown. Bar graph showing the percentage of each gene score level in 0, 1, 2, and 3 score levels of APE1. Data were categorized as follows: (i) score 0, no expression in tumor cells; (ii) score 1, faint/barely perceptible partial expression in < 10% of tumor cells; (iii) score 2, weak to moderate expression in > 10% of tumor cells; iv) score 3, strong expression in > 10% of tumor cells

Next, IHC analysis was performed in 31 NSCLC tissue specimens to examine the association between APE1 and some of its target genes identified in the above-described analysis. Representative images of APE1-high and -low examples for the NSCLC were shown in Fig. 6. Notably, APE1 overexpression was associated with DICER1 and TGM2 reduction, and similar results were obtained for FLT1 and JAG1 while APE1 accumulated (IHC = 2,3). In addition, there was no manifest correlation between APE1 and CCN2 or CDKN1A. Finally, as APE1 increased gradually, the expression level of ADM decreased first and subsequently increased. Therefore, data obtained from cancer specimens only partially recapitulated our findings using the A549 cell line model but clearly support the evidence for the existence of a direct relationship between APE1 and DICER1 expression.

### APE1 regulates DICER1 expression through miR-33a and miR-130b

We decided to focus our attention on the possible role of APE1 in the expression of DICER1 since the downregulation of DICER1 was related to EMT and tumor metastasis [64, 65].

**Fig. 7:**
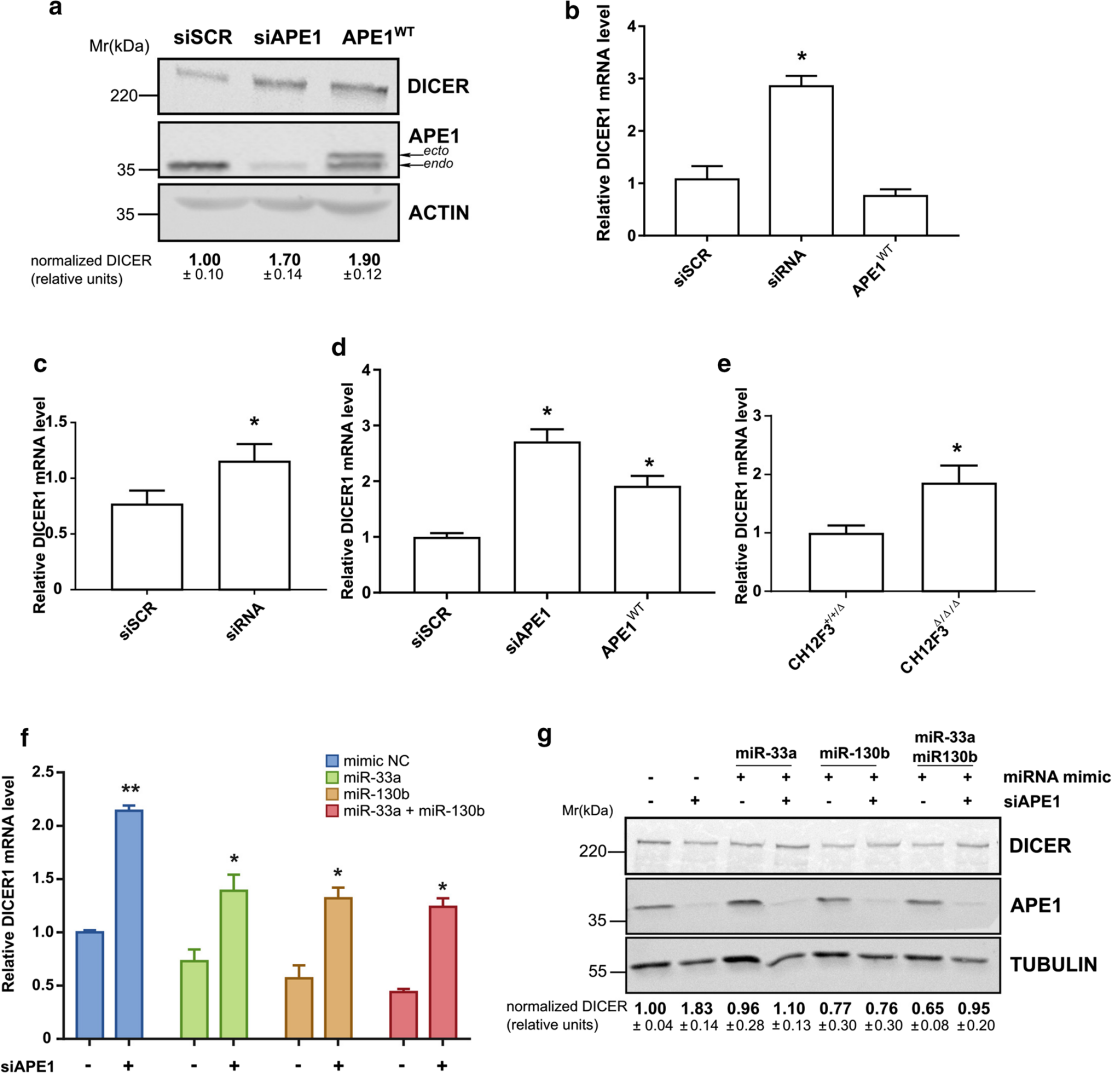
Downregulation of miR-33a and miR-130b by APE1 affects Dicer1 protein expression. **a** Dicer1 protein levels in A549 cells silenced for APE1 or transiently transfected with the expression plasmid for the FLAG-tagged siRNA-resistant APE1 protein APE1^WT^. Representative western blotting analyses on A549 total cell extracts are shown. The endogenous (*endo*) and ectopic (*ecto*) form of the APE1 protein is visible. Actin was used as a loading control and for data normalization. The amount of Dicer1 protein normalized to siSCR is reported under each lane. **b** DICER1 mRNA levels evaluated by qRT-PCR analysis in A549 cells silenced for APE1 or transiently transfected with an expression plasmid for the FLAG-tagged siRNA-resistant APE1 protein APE1^WT^. Histograms report data using the ΔΔCT method with GAPDH as the reference. **c**, **d**, **e** DICER1 mRNA expression levels were assessed by qRT-PCR and normalized with GAPDH in JHH-6 (**c**), HeLa cell clones (**d**) and CH12F3 (**e**) silenced for APE1 (see also Fig. S6). **f**, **g** A549 cells were transiently silenced for APE1 for 72 h and then transfected for 24 h with miR-33a, miR-130b mimics or negative control (mimic NC). DICER1 mRNA (**f**) and protein (**g**) expression levels were determined (see also Fig. S7). The amount of Dicer1 protein normalized to siSCR trasnfected with mimic negative control is reported under each lane. Statistical significance is represented as *p < 0.05, **p < 0.01

Based on bioinformatics analysis, the DICER1 transcript is targeted by five DE-miRNAs: miR-33a, miR-92b, miR-130b, miR-200c, and miR-218 [66–68]. In particular, previous studies showed that miR-130b overexpression empowered cell motility by targeting DICER1 expression [69] and that miR-33a had a role in regulating key EMT factors [70]. Since all these miRNAs resulted down-regulated upon APE1 silencing, we first validated, in our samples, if this impairment could affect DICER1 expression at the protein (Fig. 7A) and mRNA (Fig. 7B) levels. As evident, APE1 silencing resulted in the upregulation of both the protein and mRNA levels in accordance with the dysregulation of the above-mentioned miRNAs.

As APE1 depletion likely impaired miRNA processing, we also tested if APE1 overexpression would give the opposite effect by transfecting the A549 cells with a plasmid encoding the APE1 FLAG-tagged protein. The absence of a statistically significant effect on the Dicer1 protein and mRNA levels suggests that other proteins, in addition to APE1, may act as the rate-limiting factors (Fig. 7A, B). Additional experiments are required to establish the specific molecular mechanism involved.

Similar results on the expression of miR-33a, miR-130b, miR-200c, and miR-218 were obtained in different lung cancer cell lines (SK-MES-1, H358, H3255) (data not shown) demonstrating the general validity of our findings. The evaluation of the impaired expression of DICER1 upon APE1 dysregulation (both silencing or overexpression) was confirmed in another lung cancer cell line, the H358 cell line (Fig. S6) and cancer cell lines of different origin, i.e., JHH-6 (Fig. 7C), HeLa (Fig. 7D), as well as in non-cancer APE1-KO mouse lymphocytes (CH12F3) [27] (Fig. 7E and Fig. S6), supporting the notion that a common regulatory mechanism is possibly responsible for the regulation of the miRNA processing mechanism. Indeed, we found that among the five predicted miRNAs targeting DICER1, miR-33a resulted significantly dysregulated in all of the three tested cell lines (Fig. S6). Therefore, we concentrated on the APE1-miR-33a-DICER1 axis for further analyses.

To better characterize the involvement of APE1 in DICER1 expression through the regulation of miR-33a, we transfected miR-33a mimics in A549 cells depleted of APE1. The miR-130b mimic was also transfected since this was the only miRNA for which there is clear published evidence demonstrating its involvement in targeting DICER1 expression [69]. Furthermore, a combination of both miRNA mimics was also tested. We found that consistently with our hypothesis, DICER1 mRNA levels (Fig. 7F) and, to a less extent, its protein levels (Fig. 7G) significantly decreased when miR-33a and miR-130b mimics were transfected, compared to those transfected with mimics negative controls (Fig. S7). Different kinetics in the turnover rates between the mRNA levels and the protein levels of DICER1 can explain the discrepancies observed between mRNA and protein results. Hence, we assumed that APE1 could control DICER1 expression levels through the regulation of miR-33a and miR-130b.

### Correlation of APE1 and DICER1 expression levels in cancer specimens

**Fig. 8:**
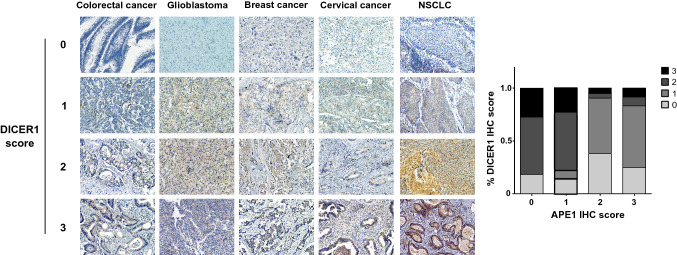
Correlative expression of APE1 and DICER1 in human cancer specimens. Dicer1 protein expression was determined by IHC assay and the representative images are shown. Bar graph showing the percentage of each score level of Dicer1 in 0, 1, 2, and 3 score level of APE1. Data were categorized as follows: (i) score 0, no expression in tumor cells; (ii) score 1, faint/barely perceptible partial expression in < 10% of tumor cells; (iii) score 2, weak to moderate expression in > 10% of tumor cells; (iv) score 3, strong expression in > 10% of tumor cells

To confirm the association of APE1 and DICER1 in cancer progression, a cohort of a hundred tissue samples from chemotherapy- and radiotherapy-naïve patients diagnosed with colorectal cancer, glioblastoma, breast cancer, cervical cancer, and non-small cell lung cancer (NSCLC) were also tested for APE1 and Dicer1 protein expression by IHC, and representative images of APE1-high and -low examples for the five cancer types are shown in Fig. 8. The statistical results of the IHC assay depict a trend showing that the tumor groups characterized by higher Dicer1 expression (IHC score = 2 or 3) showed also APE1 low expression (IHC score = 0 or 1), suggesting that APE1 and Dicer1 protein levels are inversely correlated (*r* = − 0.437, *p* < 0.0001) across the cohort.

## Discussion

Experimental and clinical data have shown that altered biogenesis of miRNAs is a common feature of chemoresistance in several cancers, including lung carcinomas, thus limiting curative effects. Therefore, elucidating the molecular mechanisms behind chemoresistance represents the primary challenge to improve the effectiveness of lung cancer treatments. Numerous research findings have shown that miRNAs are involved in drug resistance by targeting drug-resistance-related genes and genes related to the cell cycle, cell proliferation, and apoptosis [71].

Another mechanism contributing to drug resistance is represented by the alteration of the DNA damage repair capacity of tumors. Increased expression of DNA damage repair enzymes has been associated with cell resistance to DNA alkylating agents [72, 73]. In this context, the BER enzyme APE1 is considered a good predictive biomarker for lung cancer prognosis and treatment effect, since its overexpression is an important cause of poor chemotherapeutic efficacy in NSCLC patients [18, 20, 21, 29]. APE1, besides being a key DNA repair enzyme, modulates through its redox function the activity of several transcription factors related to cancer progression and metastasis [23]. Recently, we also demonstrated that APE1 actively contributes to cancer progression by controlling gene expression through its direct processing activity of specific miRNAs [25].

In this study, we first applied high-throughput approaches to identify miRNAs differentially expressed upon APE1 downregulation in the A549 cell line. Thirteen miRNAs were chosen as candidates for further analysis either for their potential prognostic value or due to their common dysregulated expression in APE1-silenced HeLa cells: 11 miRNAs resulted down-regulated (miR-24, miR-183, miR-660, miR-130b, miR-543, miR-200c, miR-376c, miR-218, miR-146a, miR-92b, and miR-33a), 2 were up-regulated (miR-4488 and miR-1246). Survival analysis confirmed the prognostic value of the thirteen-miRNAs signature in LUAD patients and, furthermore, their altered expression was confirmed in human cancer specimens, thus supporting the prognostic value of our findings.

Functional enrichment analysis on validated targets of this signature releveled *microRNAs pathway in cancer* as one of the most predicted pathways affected by the thirteen DE-miRNAs. Among the validated targets, DICER1 was the node regulated by the highest number of DE-miRNAs (i.e., miR-33a, miR-92b, miR-130b, miR-200c, and miR-218). Then, we focused our attention on the APE1-miR-33a-DICER1 axis since miR-33a expression was down-regulated also in other cell lines depleted for the APE1 protein (i.e., HeLa, JHH-6 and mouse APE1-null cells) alongside with an upregulation of DICER1 expression, suggesting the presence of a conserved co-regulatory mechanism.

miR-33a, an intronic miRNA located within the sterol regulatory element-binding protein 2 (SREBP-2) gene, is found to be dysregulated in several human cancers including melanoma [74], breast [75], and osteosarcoma [76], in which it acts as a tumor suppressor. In lung cancer, its down-regulation is predictive of a poor prognosis [77], as it is involved in EMT through the targeting of key pro-EMT genes [70, 78]. Nevertheless, its clinical significance remained elusive since other findings demonstrated opposite effects, indicating a complex and context-dependent response. For example, miR-33a is up-regulated in chemoresistant osteosarcoma [79] and, furthermore, its increased expression is a potential prognostic marker of HCC [80]. In the present study, we found a downregulation of miR-33a expression in the A549 cell line upon APE1 depletion and a significant upregulation in a cohort of NSCLC specimens, in which APE1 is overexpressed.

A global miRNAs dysregulation, matched by a defect in miRNAs production, has emerged as a hallmark of human cancer [65, 81]. Among the different mechanisms that can explain miRNAs deregulation, impairment of the miRNA processing machinery is attracting increasing interest in the field [82].

Here, we identified a global downregulation of miRNA expression upon APE1 silencing, in agreement with our previous observation [25] and, for the first time, we validated DICER1 as a direct functional target of miR-33a in the A549 cell line and confirmed a previous study showing that miR-130b directly targeted DICER1 3’UTRs [69]. DICER1 belongs to the RNase III family of double-stranded RNase, representing a key enzyme controlling the maturation of miRNAs in the cytoplasm [83]. Altered DICER1 expression has been documented in various tumors, such as breast [84], ovarian [85], colorectal [86], and lung cancers [87]. In particular, low levels of DICER1 in lung cancer are known to correlate with a poor clinical outcome [88, 89]; whereas high DICER1 expression levels entailed a significantly better prognosis [90]. However, the reasons for DICER1 downregulation in cancers are not fully understood and represent an emerging open field. Several mechanisms have been described as underlying regulators of DICER1 reduced expression, such as monoallelic loss [91] and transcriptional and epigenetic regulation [92]. Our study suggests that the post-transcriptional regulatory mechanism mediated by miRNAs can impinge DICER1 mRNA expression, as already shown for miR-107 [64]. Furthermore, DICER1 depletion and, consequently, miRNAs down-regulation, have been shown to foster epithelial-to-mesenchymal transition (EMT) and promote higher metastatic potential [64, 65]. Interestingly, all of the five APE1 DE-miRNAs involved in DICER1 targeting (i.e., miR-33a, miR-130b, miR-92b, miR-200c, and miR-218) have been also implicated in EMT processing through the regulation of key modulators, such as the transcription factors ZEB1, ZEB2, TWIST or signaling transduction pathways, implicated in EMT [79, 93–95]. In particular, a recent work demonstrated that overexpression of miR-130b promoted invasion and matrix metalloproteinase-2 (MMP-2) activity in A549 cells and, consistently with this, miR-130b expression was significantly increased in NSCLC clinical specimens from patients affected by vascular and lymphatic invasion [93]. Mounting evidence have also shown that EMT could be a mechanism rendering cell resistant to anti-cancer therapy. Likewise, the involvement of miRNAs in the combined regulation of EMT and chemoresistance is tangible. Alongside, some studies have already highlighted the association of APE1 overexpression with increased EGFR-TKI-resistant cells due to epithelial-to-mesenchymal transition mechanisms [20, 21]. However, nobody has ever linked this regulation to a possible involvement of APE1 through its activity on miRNA expression. Here, we showed that APE1 up-regulation in lung cancer positively correlates with an increased expression of miRNAs that target DICER1, thus affecting EMT-driven metastatic pathways. Moreover, this regulatory axis also involved genes associated with DNA damage and immune response. Interestingly, we confirmed the expression trends of eight EMT-related target genes also in TCGA-LUAD tumor samples, indicating that this regulatory network could indeed underline a likely contribution of APE1 in the regulation of miRNAs function in lung cancer progression, at different levels.

The detailed interplay between these regulatory pathways remains to be elucidated, as well as the molecular mechanisms responsible for the observed specific activity of APE1 on certain miRNAs. APE1-redox and -endonuclease inhibitors (Fig. 3B) only partially explained the observed miRNAs dysregulation upon APE1 silencing. miRNAs biological regulation is a complex process, typically involving an intricate network of regulatory loops. We recently characterized the APE1 interactome finding several proteins associated with miRNA binding and processing (e.g., NPM1, hnRNAPA2/B1, FUS, hnRNPD, hnRNPE1, etc.) that might explain APE1 indirect involvement in miRNA dysregulation. A preliminary network analysis was performed to elucidate this regulatory axis showing that significantly up-regulated APE1-interacting partners associated with a poor prognostic value were related to their DE-miRNA targets (Fig. S8).

The IHC analysis performed on a cohort of NSCLC patients, characterized by different expression levels of APE1, confirmed a good correlation existing in the expression of four (i.e., DICER1, TGM2, FLT1, and JAG) out of seven genes, hypothetically regulated by APE1 through specific miRNAs, except for CCN2, CDKN1A, and ADM. These data, while showing a limitation of our overall approach, are ascribable to the biological complexity of tumor development in vivo with respect to the mechanisms acting in in vitro cell cultures.

In summary, our results revealed another layer of gene regulation in the APE1-associated gene expression axis, which could provide a better understanding of the interaction between mRNAs and miRNAs. Our results identify, for the first time, a crucial role for the miR-33a/miR-130b-APE1-DICER1 axis in NSCLC progression. The work developed herein enabled us to evaluate APE1 contribution to lung cancer progression and metastasis, identifying candidate miRNAs, playing a pivotal role in these processes. We acknowledge that our results represent a preliminary hypothesis, which should be experimentally validated through additional in vivo studies. Our data suggest that modulating the expression levels of APE1 may affect miRNA expression and, therefore, clinical responses to anticancer drug treatments. We propose the use of APE1-regulated miRNAs as novel prognostic biomarkers that could be potentially relevant to develop innovative RNA-based drugs for targeting oncogenes, in multiple cancers, in combination with APE1 inhibitors. Further exploration of the recognized associations is expected to improve drug effectiveness and to identify interesting therapeutical combinations for precision medicine.

## Supplementary Information

Below is the link to the electronic supplementary material.

Supplementary file1 (XLSX 80 KB)

Supplementary file2 (XLSX 10265 KB)

Supplementary file3 (DOCX 12 KB)

Supplementary file4 (XLSX 75 KB)

Supplementary file5 (DOCX 13 KB)

Supplementary file6 (DOCX 14 KB)

Supplementary file7 (DOCX 2859 KB)

## Data Availability

Raw data corresponding to RNA-seq and NanoString experiments are uploaded with GEO Superseries accession GSE166750. For reviewers, to access the data, go to https://www.ncbi.nlm.nih.gov/geo/query/acc.cgi?acc=GSE166750 Enter token ebqrkkeyrrazfob into the box. For the individual experiments: NanoString (GEO accession GSE166749) https://www.ncbi.nlm.nih.gov/geo/query/acc.cgi?acc=GSE166749 (token wdobkayilraxhaz); RNA-seq (GEO accession GSE166664) https://www.ncbi.nlm.nih.gov/geo/query/acc.cgi?acc=GSE166664 (token szotsyuoftcxlmn).

## Abbreviations

APE1: Apurinic/apyrimidinic endodeoxyribonuclease 1
BER: Base excision repair
DE-miRNA: Differentially expressed miRNA
EMT: Epithelial-to-mesenchymal transition
NSCLC: Non-small cell lung carcinoma
PPI: Protein–protein interaction
