# Supplementary material for: APE1 controls DICER1 expression in NSCLC through miR-33a and miR-130b
Source: Cell Mol Life Sci. 2022 Jul 25;79(8):446. doi: 10.1007/s00018-022-04443-7 (PMC9314295; doi:10.1007/s00018-022-04443-7)
Supplement: Supplementary file 3 — Supplementary file3 (DOCX 12 KB) [file 18_2022_4443_MOESM3_ESM.docx]

| Variables | Univariate Analysis | | Multivariate analysis | |
| --- | --- | --- | --- | --- |
|  | Hazard ratio (95% CI) | P value | Hazard ratio (95% CI) | P value |
| Age | 1 (0.99-1) | 0.73 | 1 (0.98-1.01) | 0.66 |
| Tumor stage | 1.5 (1.2-1.8) | < 0.001 *** | 1.38 (1.10-1.73) | 0.006 ** |
| Distant metastasis (yes/no) | 2.2 (1.3-4) | 0.007 ** | 1.55 (0.83-2.91) | 0.17 |
| Gender (male/female) | 1 (0.76-1.4) | 0.8 | 0.90 (0.61-1.34) | 0.62 |
| miRNA sign PI  (high risk/low risk) | 1.86 (1.3-2.7) | < 0.001 *** | 1.97 (1.25-3.11) | 0.004 ** |

**Univariate and multivariate regression analysis of lung adenocarcinoma (TCGA-LUAD)**
