# Supplementary material for: APE1 controls DICER1 expression in NSCLC through miR-33a and miR-130b
Source: Cell Mol Life Sci. 2022 Jul 25;79(8):446. doi: 10.1007/s00018-022-04443-7 (PMC9314295; doi:10.1007/s00018-022-04443-7)
Supplement: Supplementary file 5 — Supplementary file5 (DOCX 13 KB) [file 18_2022_4443_MOESM5_ESM.docx]

**List of primary and secondary antibody used for western blot**

| **Antigen** | **Diluition** | **Company** |
| --- | --- | --- |
| APE1 | 1:2000 | Novus, NB 100-116 |
| DICER1 | 1:500 | Abcam, ab14601 |
| Tubulin | 1:2000 | Sigma, T0198 |
| Actin | 1:2000 | Sigma, A2066 |
| Anti-mouse IgG IRDye 800 | 1:10000 | LI-COR, GmbH, Germany |
| Anti-rabbit IgG IRDye 680 | 1:10000 | LI-COR, GmbH, Germany |
