# Supplementary material for: APE1 controls DICER1 expression in NSCLC through miR-33a and miR-130b
Source: Cell Mol Life Sci. 2022 Jul 25;79(8):446. doi: 10.1007/s00018-022-04443-7 (PMC9314295; doi:10.1007/s00018-022-04443-7)
Supplement: Supplementary file 6 — Supplementary file6 (DOCX 14 KB) [file 18_2022_4443_MOESM6_ESM.docx]

**List of taqman probes used**

| **Target** | **Probe** | **Company** |
| --- | --- | --- |
| hsa-miR-183-5p | 477937_mir | Thermofisher |
| hsa-miR-130B-3p | 477840_mir | Thermofisher |
| hsa-miR-4488 | 478906_mir | Thermofisher |
| hsa-miR-543 | 478155_mir | Thermofisher |
| hsa-miR-376c-3p | 478459_mir | Thermofisher |
| hsa-miR-200c-3p | 478351_mir | Thermofisher |
| hsa-miR-1246 | 477881_mir | Thermofisher |
| hsa-miR-33a-5p | 478347_mir | Thermofisher |
| hsa-miR-92b-3p | 477823_mir | Thermofisher |
| hsa-miR-218-5p | 477977_mir | Thermofisher |
| hsa-miR-146a-5p | 478399_mir | Thermofisher |
| hsa-miR-24-3p | 477992_mir | Thermofisher |
| hsa-miR-660-5p | 478192_mir | Thermofisher |
| hsa-miR-16-5p | 477860_mir | Thermofisher |
|  |  |  |

**List of primers used**

| **Target** | ***Forward (5’to 3’)*** | ***Reverse (5’to 3’)*** |
| --- | --- | --- |
| DICER1 | CACTGGCTCAGGGAAGACATT | CAACCTGGTTTGCAGAGTTGAC |
| BIRC3 | CCAAGTGGTTTCCAAGGTGT | TGGGCTGTCTGATGTGGATA |
| BRCA1 | GGTGGTACATGCACAGTTGC | ACTCTGGGGCTCTGTCTTC |
| CDK6 | TGCACAGTGTCACGAACAGA | ACCTCGGAGAAGCTGAAAC |
| CDKN1A | GAAGGTAGAGCTTGGGCAGG | GCGACTGTGATGCGCTAATG |
| GADD45A | TCAGCGCACGATCACTGTC | CCAGCAGGCACAACACCAC |
| HIST13B | ATGGCTCGTACTAAACAGAC | AGTCTTGGGCGATTTCTCG |
| STAT1 | CAGCTTGACTCAAAATTCCTGGA | TGAAGATTACGCTTGCTTTTCCT |
| ZMAT3 | AGCCTGCAGCTACTCCAGTT | AAGGAGGCATCACAGAGCTT |
| CDH1 | CCCACCACGTACAAGGGTC | CTGGGGTATTGGGGGCATC |
| VIM | ACTACGTCCACCCGCACCTA | CAGCGAGAAGTCCACCGAGT |
| ZEB1 | TGGCGGTAGATGGTAATG | GGAAGACTGATGGCTGAA |
| ZEB2 | GAGGAAGACAATATGGAAGATG | TTCAAGCAGGTAACAATACTAC |
| SNAIL1 | CGGAAGCCTAACTACAGCGA | AGAGTCCCAGATGAGCATTGG |
| TWIST | ATTCAAAGAAACAGGCGTGG | CAGAGGTGTGAGGATGGTGC |
| BIRC5 | ACCGCATCTCTACATTCAAG | CAAGTCTGGCTCGTTCTC |
| GAPDH | CCCTTCATTGACCTCAACTACATG | TGGGATTTCCATTGATGACAAGC |
