# Supplementary material for: APE1 controls DICER1 expression in NSCLC through miR-33a and miR-130b
Source: Cell Mol Life Sci. 2022 Jul 25;79(8):446. doi: 10.1007/s00018-022-04443-7 (PMC9314295; doi:10.1007/s00018-022-04443-7)
Supplement: Supplementary file 7 — Supplementary file7 (DOCX 2859 KB) [file 18_2022_4443_MOESM7_ESM.docx]

**APE1 controls DICER1 expression in NSCLC through miR-33a and miR-130b**

Giulia Antoniali^1^, Emiliano Dalla^1^, Giovanna Mangiapane^1^, Xiaolong Zhao^2^, Yi Cheng^2^, Veronica De Sanctis^3^, Dilara Ayyildiz^1^, Silvano Piazza^4,5^, Mengxia Li^2,*^and Gianluca Tell^1,^*

^1^ Laboratory of Molecular Biology and DNA repair, Department of Medicine, University of Udine, Udine, Italy;

^2^ Cancer Center of Daping Hospital, Third Military Medical University, Chongqing, China;

^3^Next Generation Sequence Facility, Department CIBIO, University of Trento, Trento, Italy;

^4^ Bioinformatics Core Facility, Department CIBIO, University of Trento, Trento, Italy;

^5^ Computational Biology, International Centre for Genetic Engineering and Biotechnology, ICGEB, Trieste, Italy.

*Corresponding authors: Gianluca Tell ([gianluca.tell@uniud.it](mailto:gianluca.tell@uniud.it)) and Mengxia Li ([mengxia.li@outlook.com](mailto:mengxia.li@outlook.com))

**Supplementary Information**

**List of content:**

**Pages 2 to 12, Supplementary Figures;**

**Page 13, Supplementary Tables;**

**
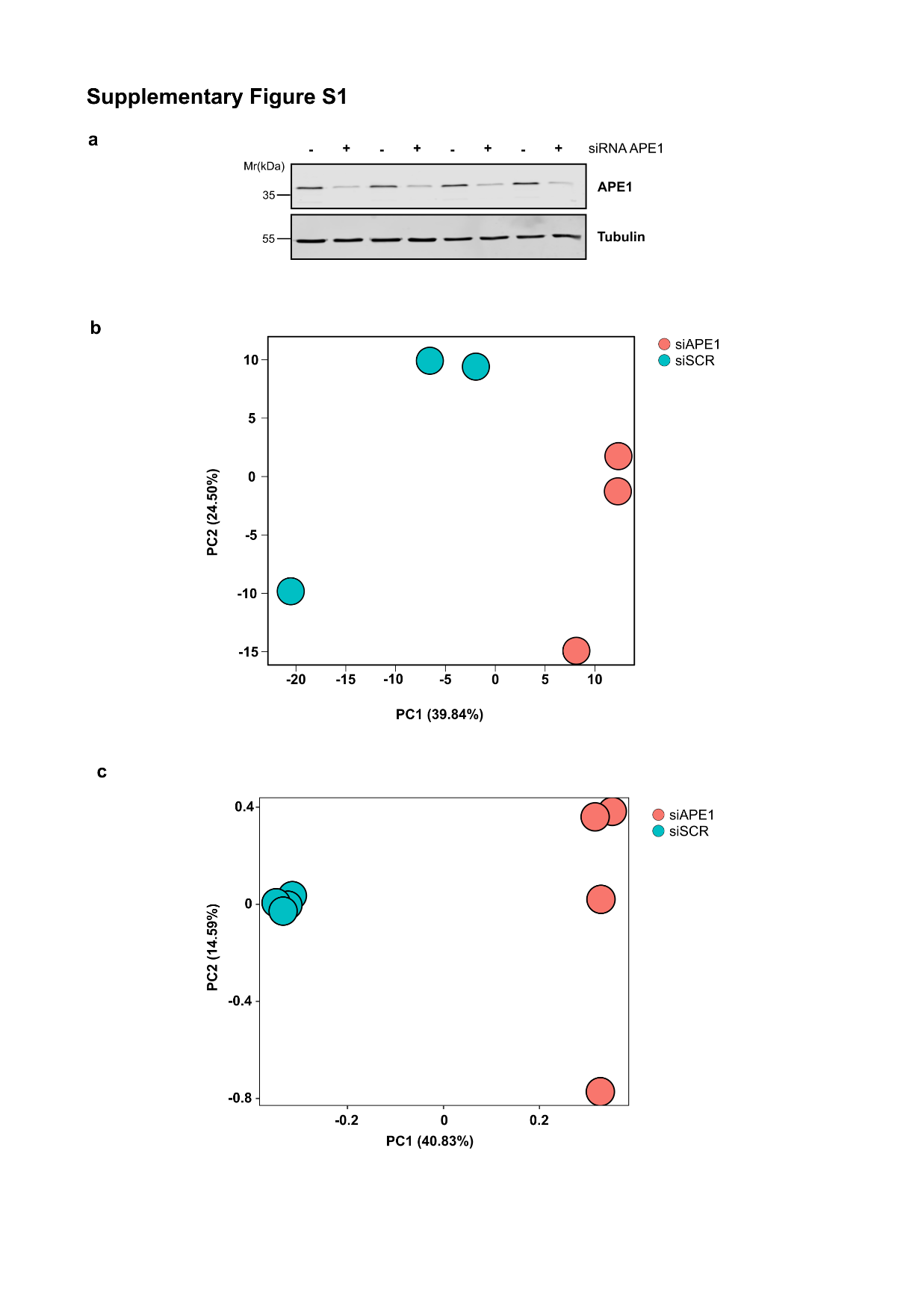
Supplementary Figures**

**Supplementary Figure 1. Global profiling of microRNA expression in A549 cells APE1-depleted.**

**a.** Representative Western blotting analyses of A549 total cell extracts silenced with APE1 siRNA probed with APE1 antibody. Tubulin was used as loading control and for data normalization. **b.** Unbiased PCA was performed on normalized expression data of all profiled miRNAs derived from Nanostring analysis. Red: siAPEX1 samples; green: control samples. Please note the first principal component, that is describing the difference between the two classes of samples, is able to explain almost 40% of the variation of the data. **c.** Unbiased PCA was performed on normalized expression data from the top one thousand genes with greater variance. Red: siAPEX1 samples; green: control samples. Please note the first principal component, that is describing the difference between the two classes of samples, is able to explain more than 40% of the variation of the data.

**
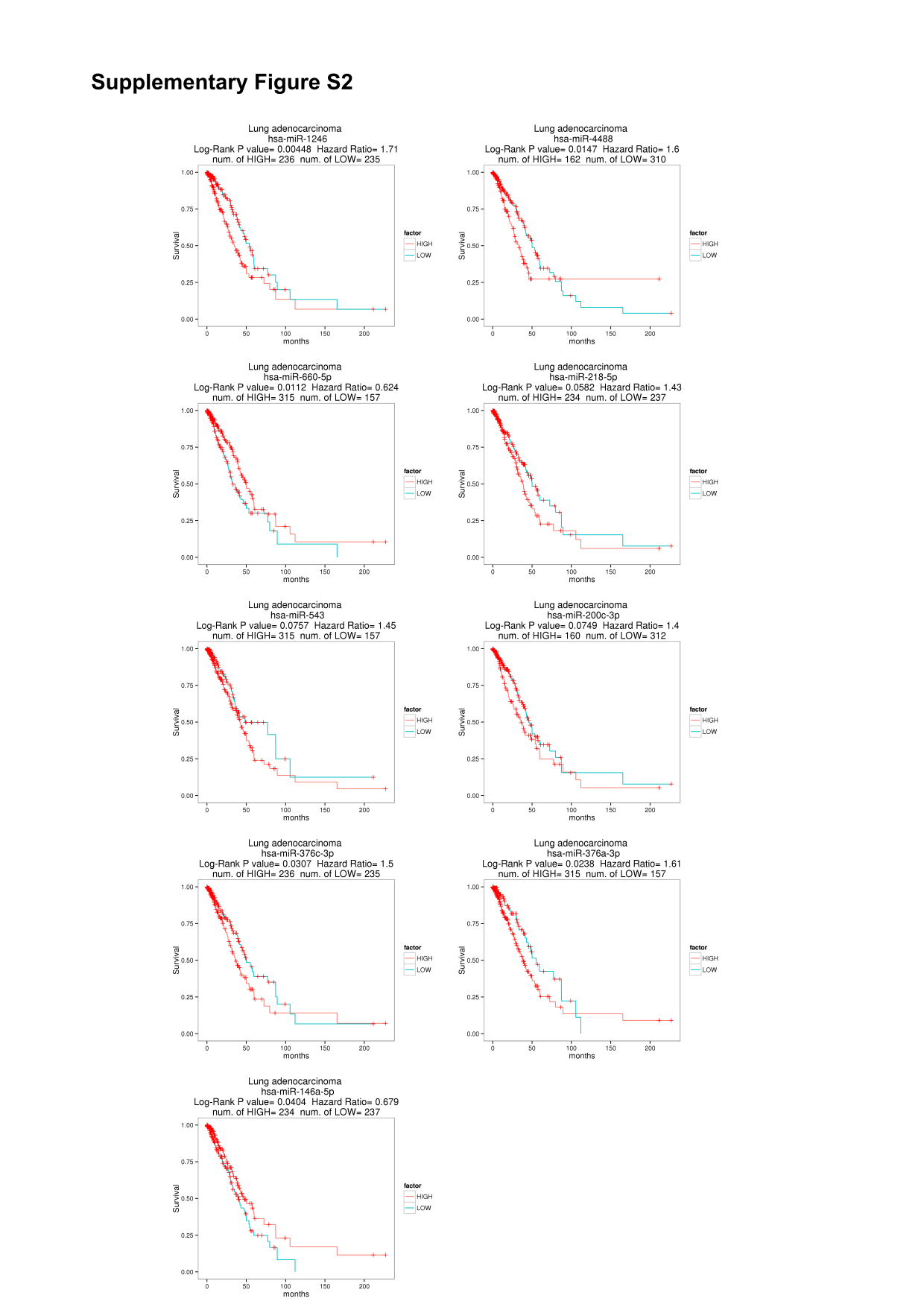
**

**Supplementary Figure 2. Prognostic value of nine A549 DE-miRNAs.** Kaplan-Meier plots showing the different overall survival rate of patients with high or low expression of selected DE-miRNAs. Patients were stratified by median, upper- or lower-tertile expression levels. Plots produced using the YM500v3 webtool.

**
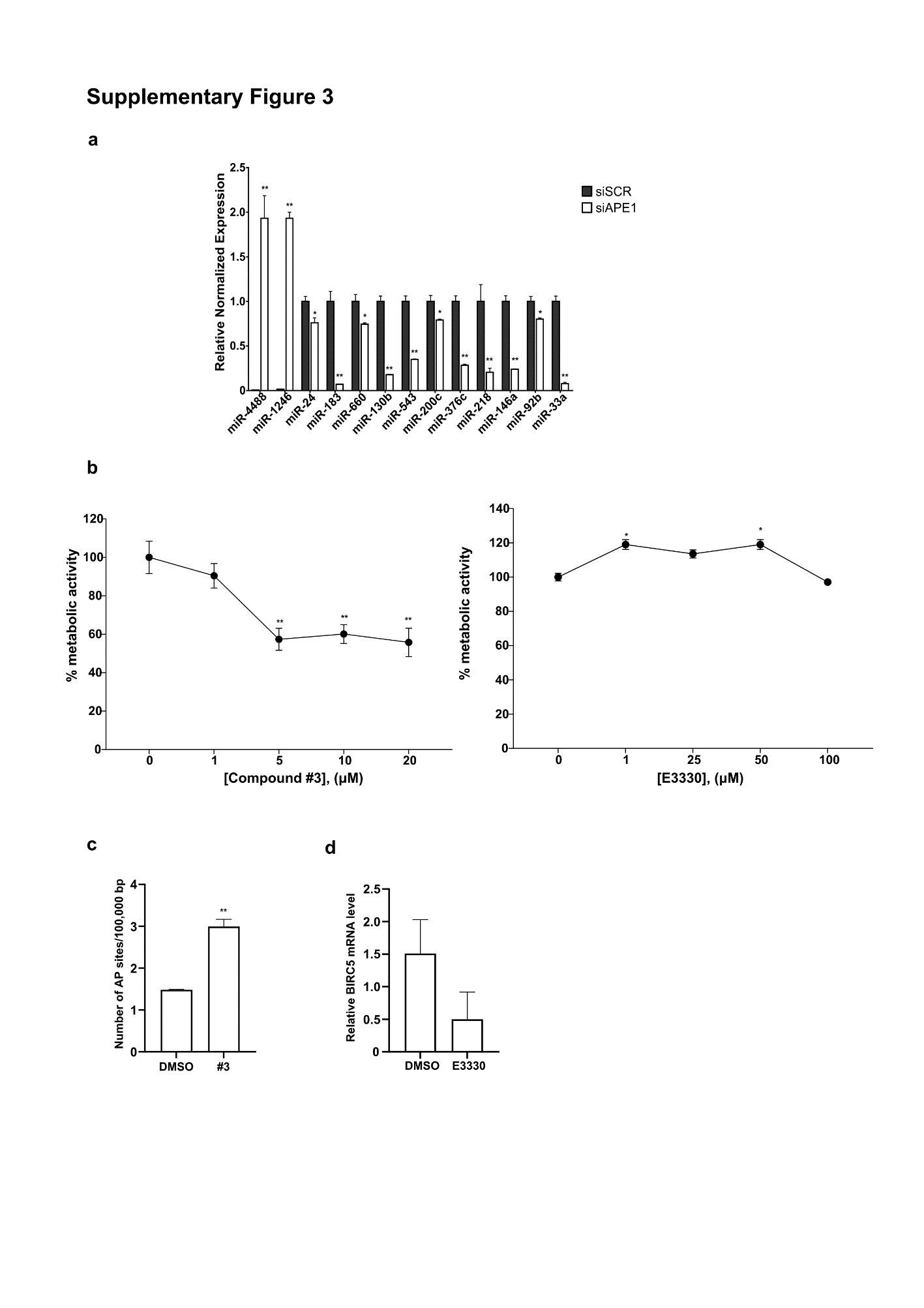
**

**Supplementary Figure 3. Validation of DE-miRNAs in cell lines.**

**a.** RT-qPCR was used to validate high-throughput data using the ΔΔCT method with miR-16-5p as the reference. Analysis was performed on pooled samples derived from the replicates used for the high-throughput analysis. **b.** MTS assay on the A549 cell line treated with APE1 inhibitors. Cells were treated with increasing concentrations of APE1 inhibitors as indicated, compound #3 and E3330 for 24 h. Untreated cells were challenged with DMSO 0.25% in RPM1 as a vehicle. In the graph, the percentage of metabolic activity with respect to DMSO-untreated cells arbitrarily set to 100% is reported. Values are mean±SD (n=3). **c.** Inhibition of the APE1 endonuclease activity was measured as an accumulation of unrepaired abasic sites (AP). Accumulation of genomic abasic (AP) lesions after compound #3 treatment (20 µM, 24 h) of A549 cells as measured by an aldehydereactive probe. Counting at the AP sites was performed by using the AP-site quantification kit, as described in Methods section. In the histogram, data are expressed as the number of AP sites per 10^5^ base pairs and represent the mean ± SD of three independent experiments. Asterisks represent a significant difference between the two conditions (untreated with DMSO vehicle and compound #3-treated cells). **d.** Inhibition of the APE1-redox activity was measured as downregulation of the expression level of the BIRC5 mRNA by qRT-PCR analysis. A549 cells were treated with E3330 (100 µM, 24 h) and DMSO 0.25% in RPM1 as vehicle. Histograms report data using the ΔΔCT method with GAPDH as the reference.

**
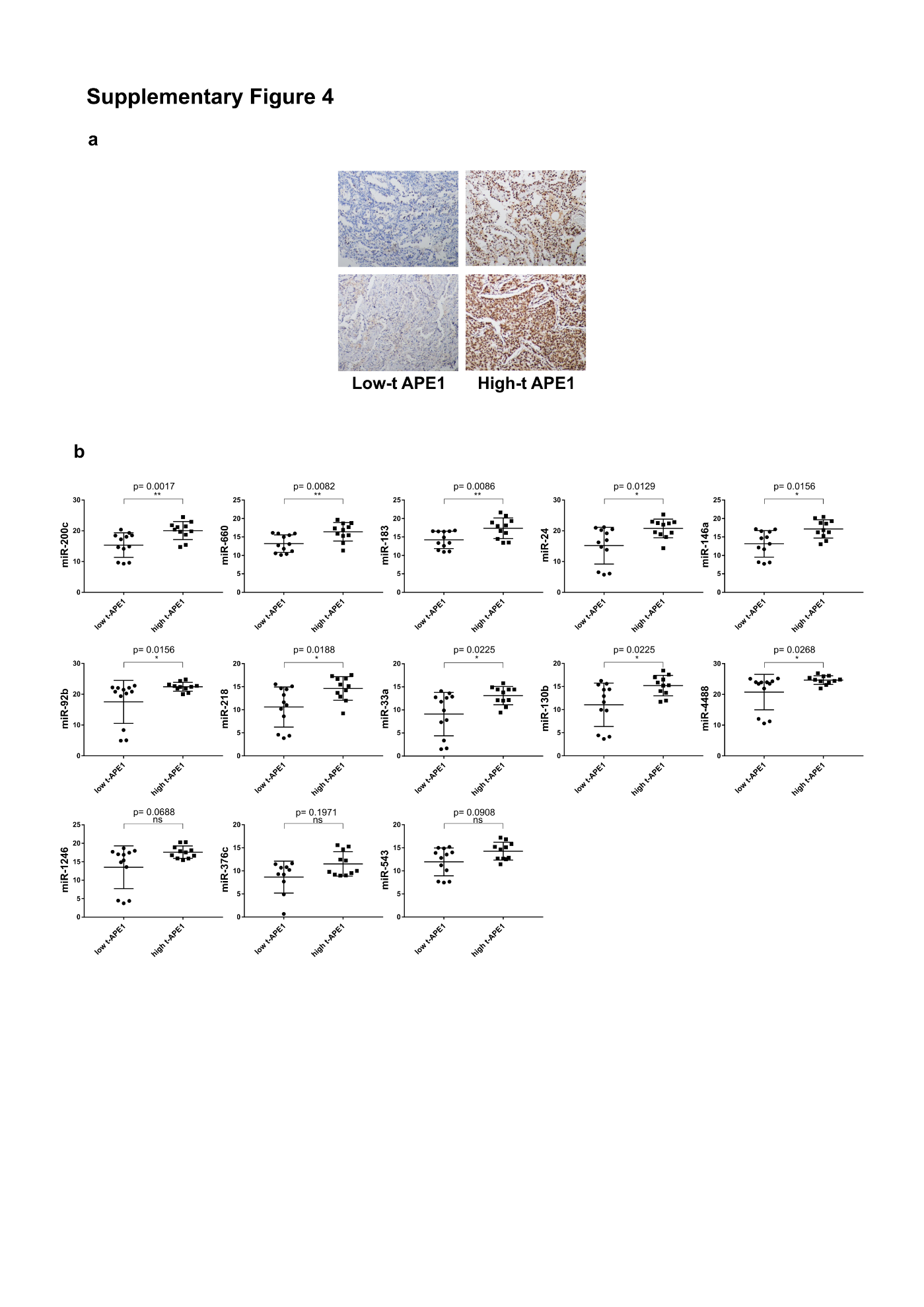
**

**Supplementary Figure 4. Validation of DE-miRNAs in human cancer specimens.**

**a.** The APE1 protein expression was determined by IHC assay and the representative images of the two cohorts were shown. Accordingly to APE1 staining, two cohorts of APE1 low- and high-expressing protein level were identified. **b.** Scatter plots report for each DE-miRNA the expression level detected in the two cohorts of low- and high- APE1 protein (score 3).

**
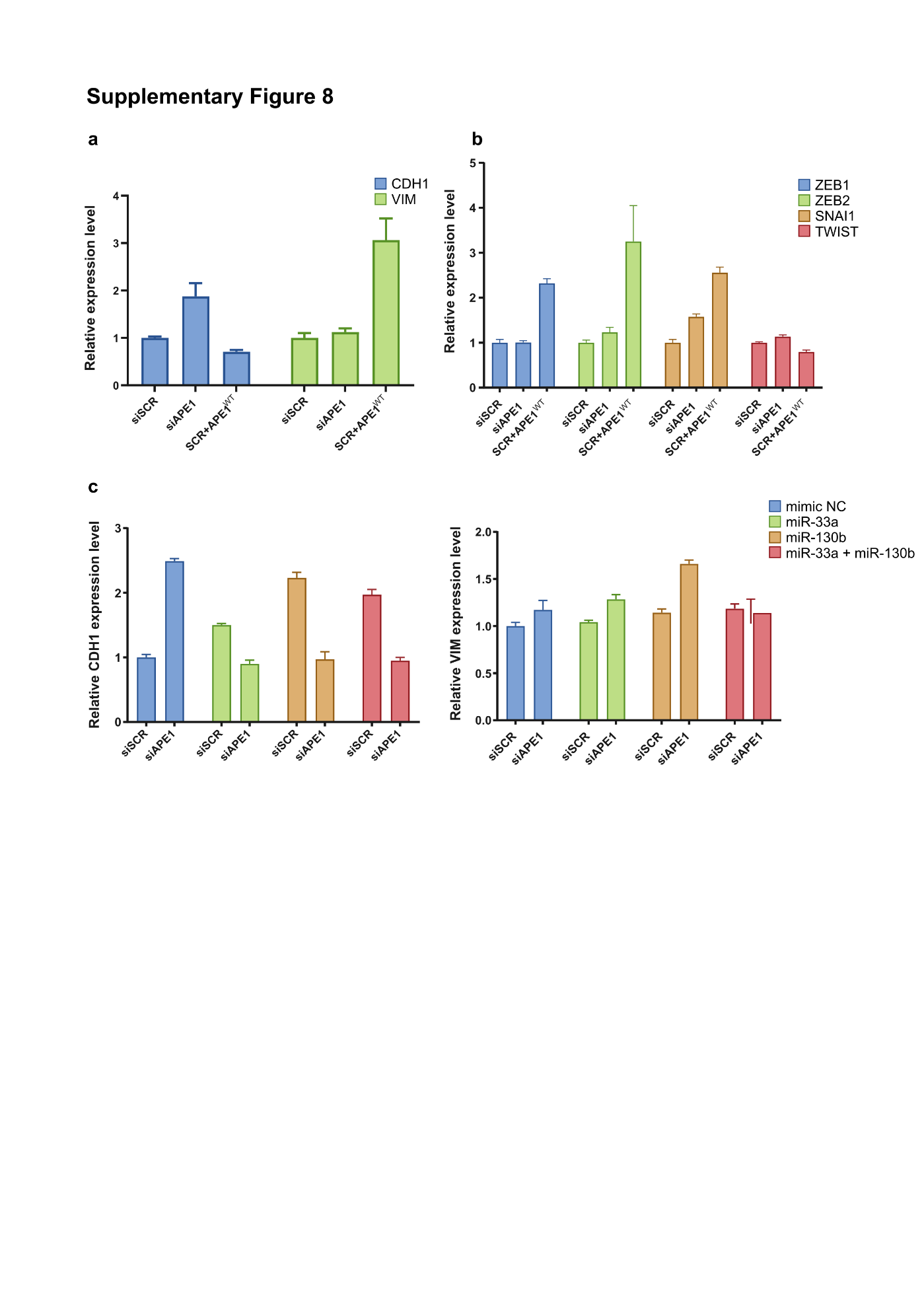
Supplementary Figure 5. APE1 promotes EMT**

**a,b.** RT-qPCR analysis of the epithelial marker E-cadherin (CDH1), mesenchymal marker vimentin (VIM) (a) and EMT transcription factors (b) were evaluated in A549 cells silenced for APE1 or transiently transfected with the expression plasmid for the FLAG-tagged siRNA-resistant APE1 protein APE1^WT^ (SCR + APE1^WT^). Histograms report data using the ΔΔCT method with GAPDH as the reference.

**
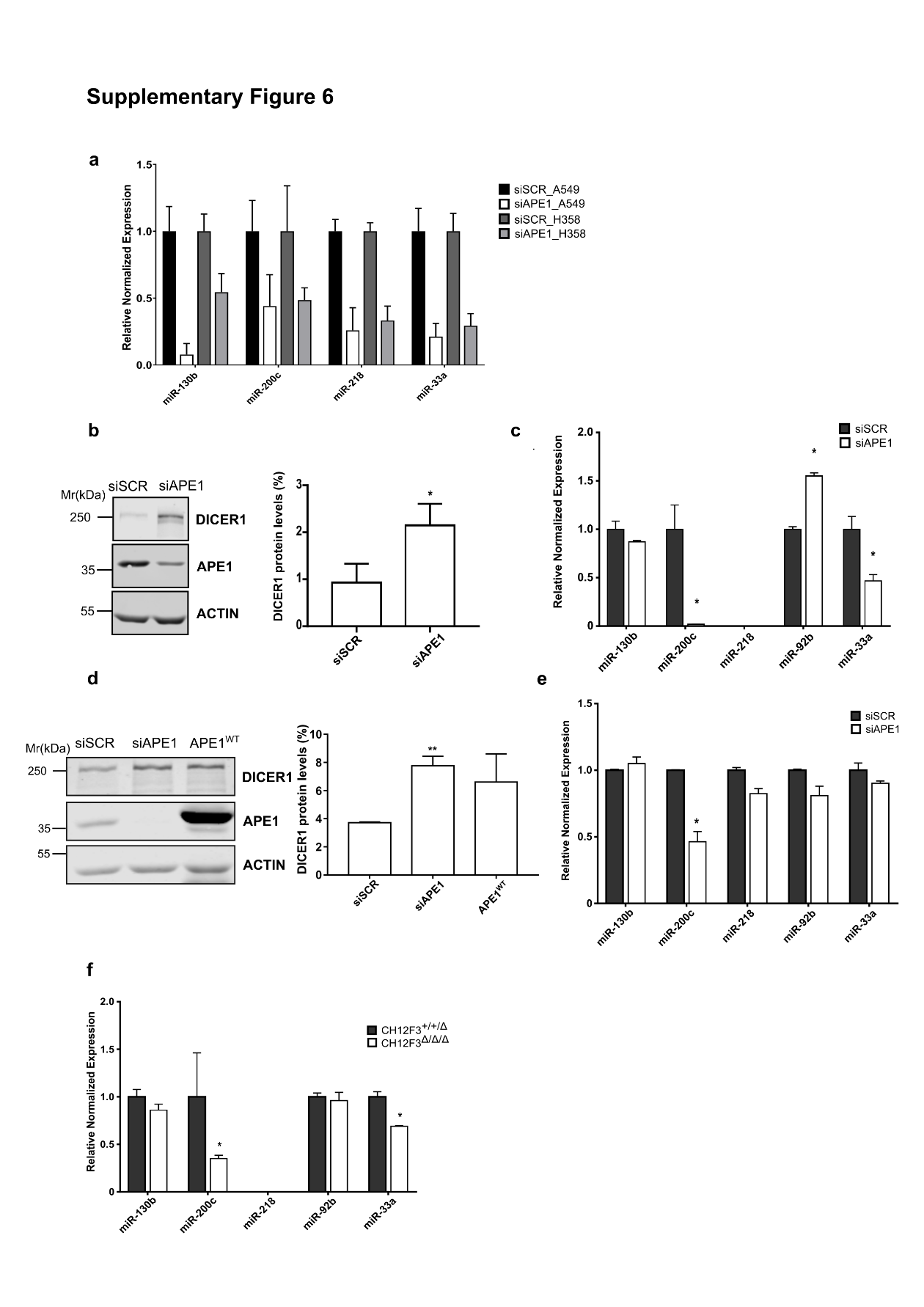
Supplementary Figure 6.** **Dicer1 protein levels are inversely correlated with APE1 in JHH-6, HeLa clones and APE1-null CH12F3 cells.**

**a.** RT-qPCR analysis of the five DE-miRNAs targeting DICER1 in A549 and H358 lung cancer cells silenced for APE1. Histograms report data using the ΔΔCT method with miR-16-5p as the reference  **b.** The JHH-6 cell line was APE1 transiently silenced for 48h and Dicer1 protein levels were evaluated by Western blot. A representative image of three independent experiments is shown. Histograms report the quantification of Dicer1 level normalized to that of actin. **c.** RT-qPCR analysis of the five DE-miRNAs targeting DICER1 in JHH-6 cells silenced for APE1. Histograms report data using the ΔΔCT method with miR-16-5p as the reference. **d.** Dicer1 protein levels were evaluated by Western blot in HeLa cell clones in which APE1 downregulation was induced by doxycycline. 9 days after induction, cells were transfected with the FLAG-tagged, siRNA-resistant APE1 wild type form (APE1^WT^) to reconstitute APE1 expression. A representative image of three independent experiments is shown. Histograms report the quantification of Dicer1 level normalized to that of actin. **e.** RT-qPCR analysis of the five DE-miRNAs targeting DICER1 in HeLa cell clones. Histograms report data using the ΔΔCT method with miR-16-5p as the reference. **f.** RT-qPCR analysis of the five DE-miRNAs targeting DICER1 in CH12F3 APE1^+/+/Δ^ and APE1 ^Δ/ Δ/Δ^ cells. Histograms report data using the ΔΔCT method with miR-16-5p as the reference. Asterisks represent a significant difference with respect to control. * p<0.05, ** p<0.001.

**
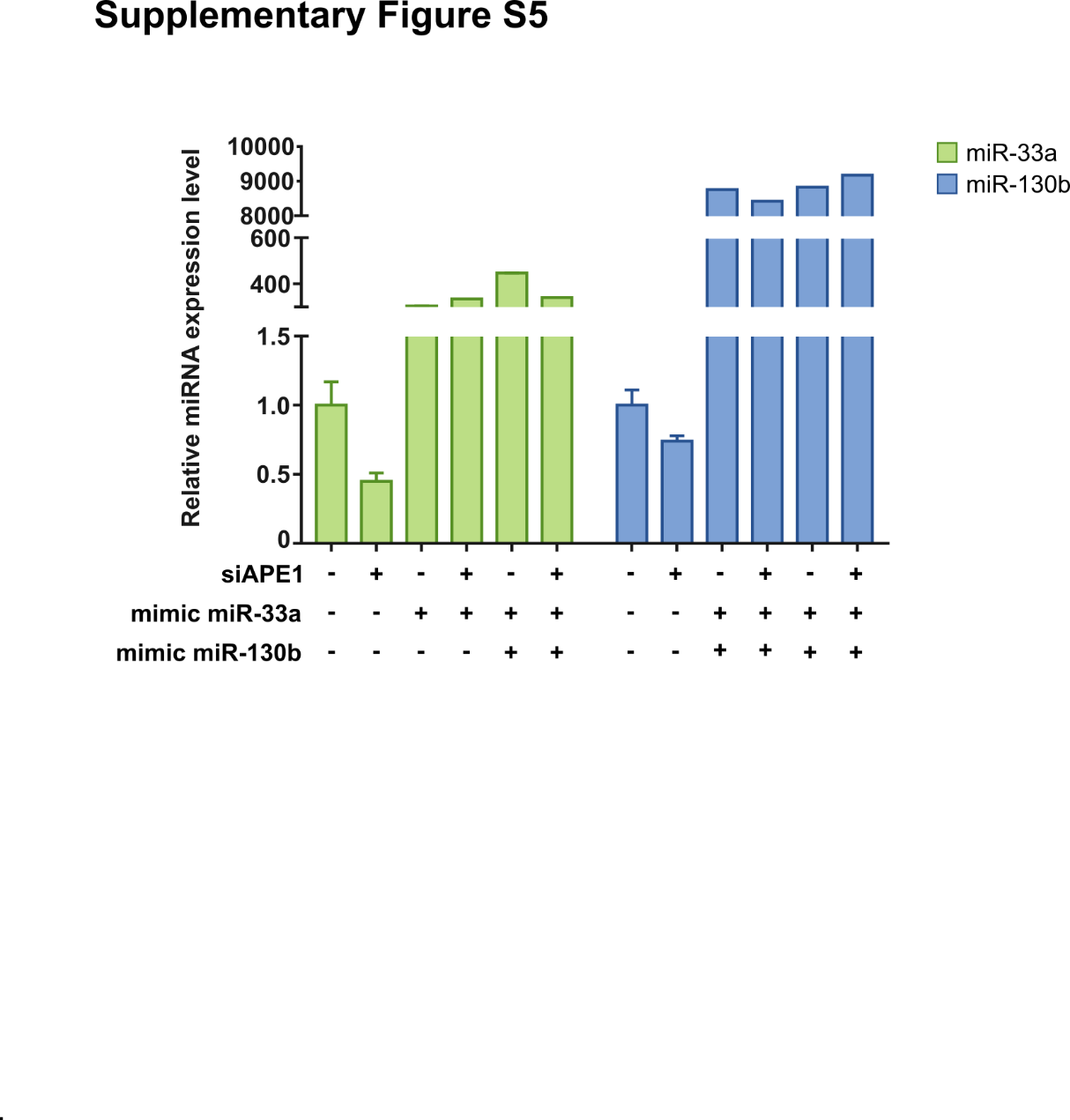
**

**Supplementary Figure 7. miRNA expression levels in A549 APE1-depleted cells transfected with miRNA mimics.**

RT-qPCR was used to validate the overexpression of miR-33a and miR130b mimics transfected in A549 cells. Histograms report data using the ΔΔCT method with miR-16-5p as the reference. Analysis was performed on A549 cells silenced for APE1.

**
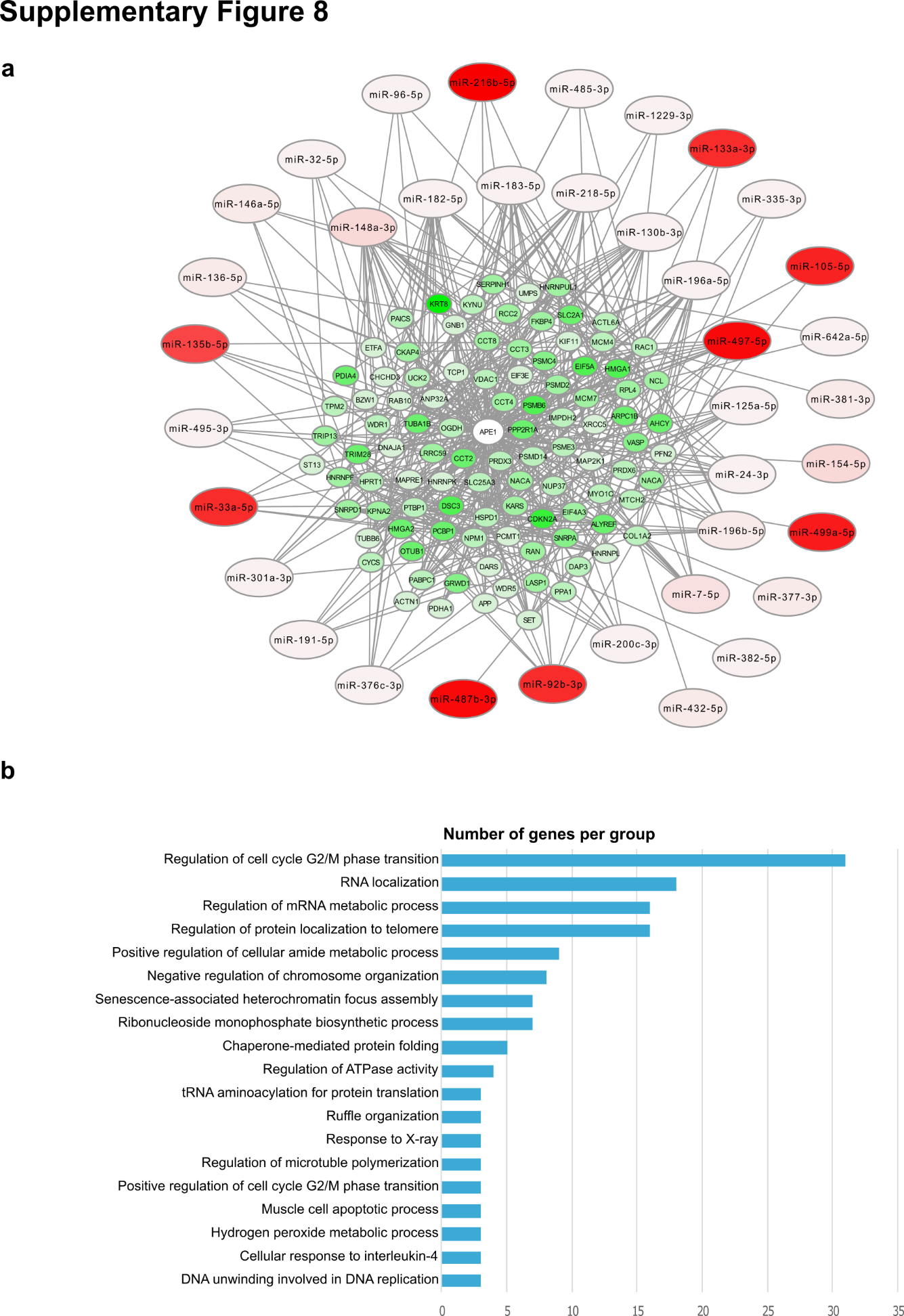
**

**Supplementary Figure 8. DE-miRNAs – APE1 PPI targets network in the TCGA-LUAD dataset.**

To explore the prognostic value of all the APE1-regulated miRNAs in lung adenocarcinoma (LUAD), the complete DE-miRNAs signature identified in our high-throughput experiments was used to study its relationship with APE1 and its interacting protein partners gene expression in LUAD dataset. **a**. The up-regulated and poor prognostic APE1 protein partners and DE-miRNAs from both NanoString and RNA-Seq experiments are represented in the network. This network, composed of 143 nodes (96 protein targets, 42 miRNAs and 5 intermediate proteins) and 599 edges, allowed the identification of a potentially significant protein community in LUAD for understanding the role of APE1 and its partners in the disease. Interestingly, miR-33a is among the miRNAs regulated by APE1 associated with a bad prognosis.

Green nodes represent up-regulated APE1 protein partners (darkest green: highest logFC; lightest green: lowest logFC) and red nodes representing DE-miRNAs (darkest red: lowest logFC; lightest red: highest logFC). DE-miRNAs surrounding the proteins were ordered clockwise according to the number of proteins they target. The APE1 protein is represented in white at the center of the network. **b.** Functional enrichment analysis of the APE1 protein partners (green nodes) that are targeted by DE-miRNAs in the network. Network proteins and DE-miRNAs were analysed with the Cytoscape plugin ClueGO to understand their role in biological processes. The majority of the interactors substantially contributed to processes mainly related to cell cycle, RNA localization and mRNA metabolism, definitely confirming APE1 role in lung cancer progression.

**
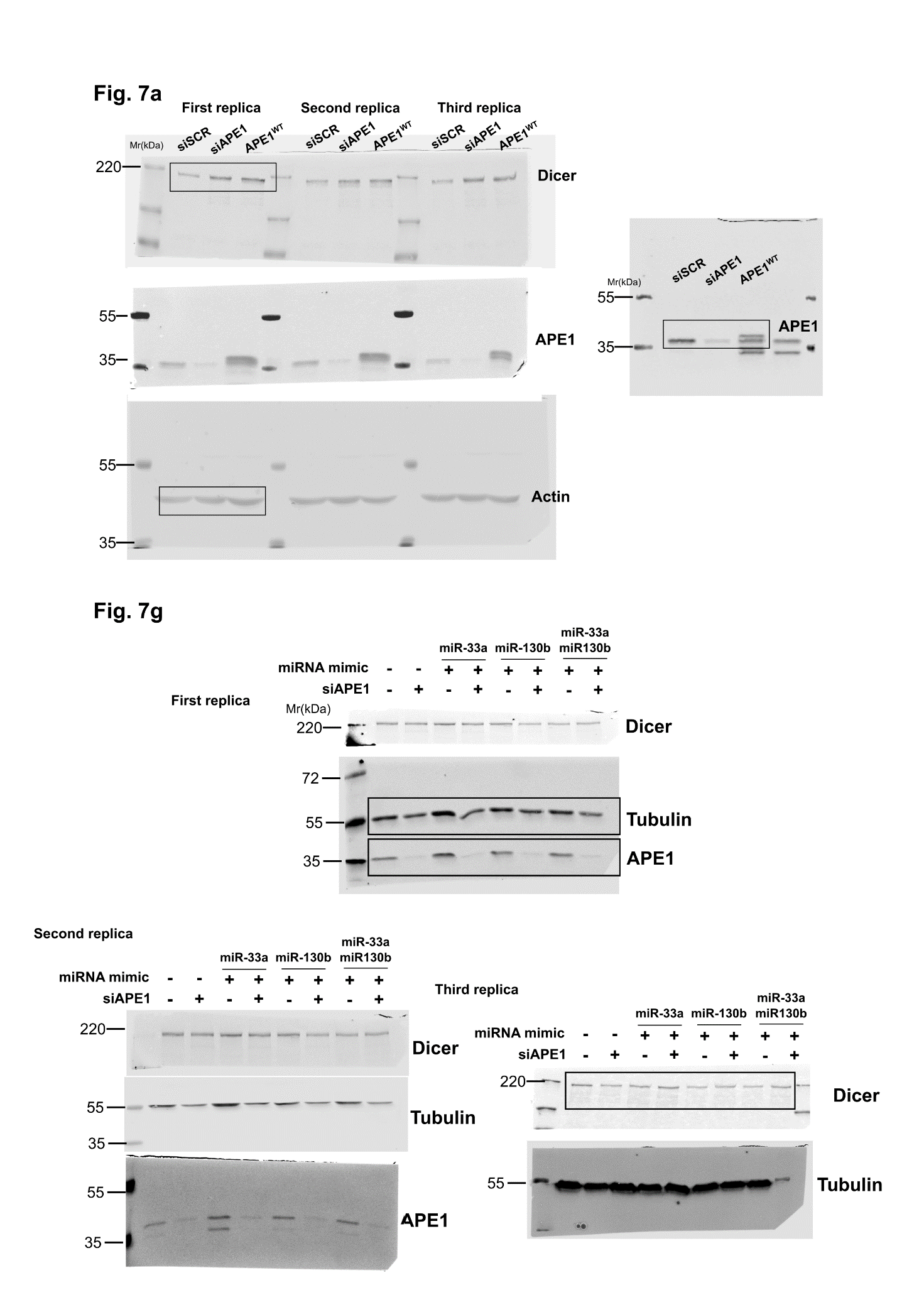
**

**Supplementary Figure 9. Uncropped blots for Figure 7.**

**
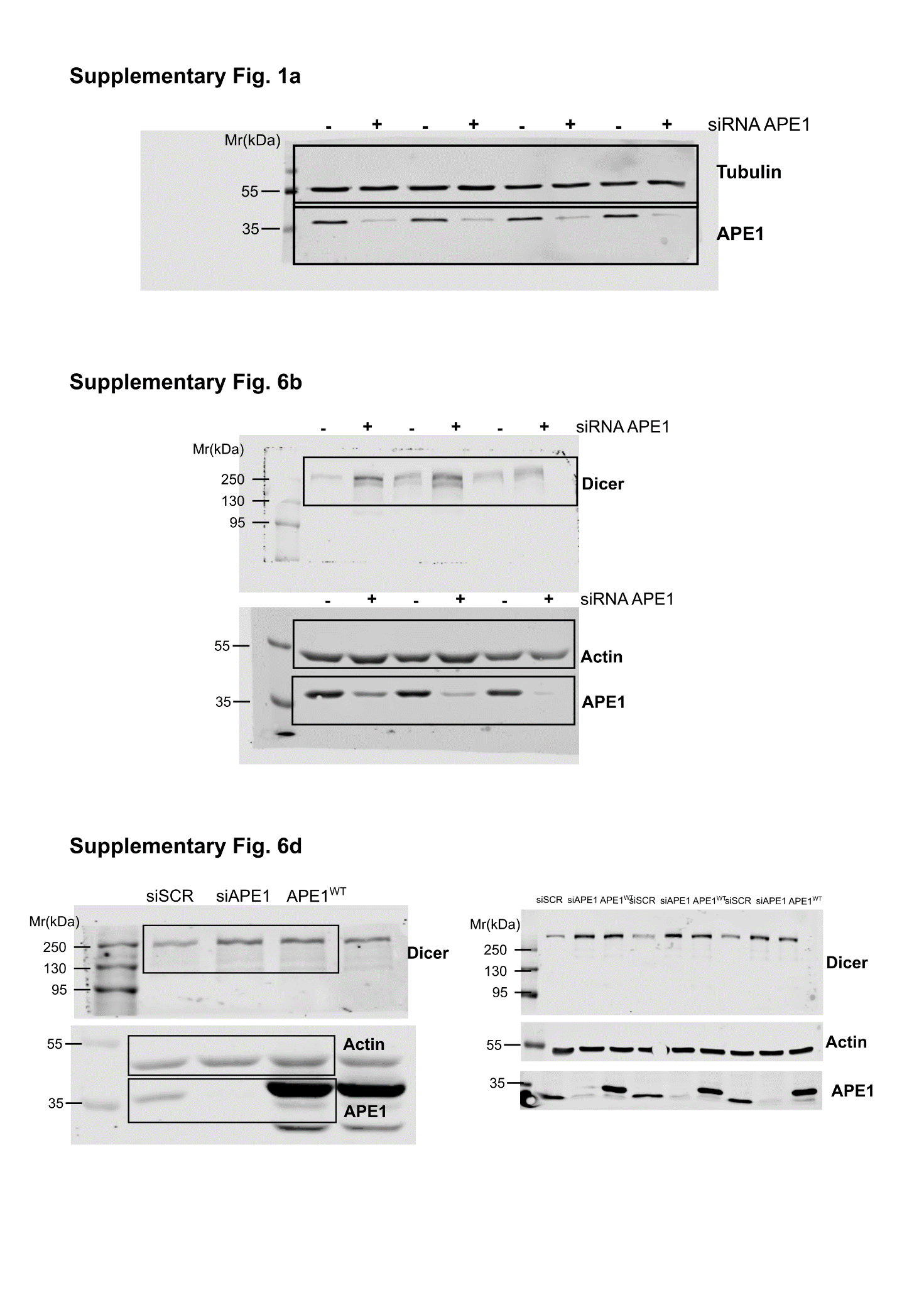
Supplementary Figure 10. Uncropped blots for Supplementary Fig S1 and S6.**

**Supplementary Tables**

**Table S1. NanoString miRNA profiling.**

Worksheet “Whole” contains the expression data of the 798 profiled miRNAs. Worksheets “q≤0.05” and “q≤0.1” contain results filtered applying two different q-value thresholds for statistical significance of differential expression. Worksheet “q≤0.1,abs(logFc)≥1” contain the final list of differentially expressed miRNAs (n=61), filtered also on the basis of differential expression with abs(logFc) ≥1.

**Table S2. RNA-seq miRNA profiling.**

Worksheet “Whole” contains the whole expression data. Worksheet “smRNA” contains results filtered applying an FDR≤0.1 threshold for statistical significance of differential expression for the smRNA features, as described by the EnsEMBL gene annotation.

**Table S3. Univariate and multivariate regression analysis of lung adenocarcinoma (TCGA-LUAD).**

Prognostic potential of the examined variables. The miRNA signature could be used as an independent predictor, after adjusting for age, gender, tumor stage and distant metastasis stage, according to the multivariate Cox regression analysis.

**Table S4. KEGG-PathwayConnector and REVIGO functional enrichment analysis of DE-miRNAs validated targets.**

Worksheet “KEGG-PathwayConnector_all” shows results sorted by increasing pvalue; worksheet “REVIGO_all” shows results sorted by increasing dispensability score; worksheet “KEGG-PathwayConnector_expressed” shows results sorted by increasing pvalue. Top10 terms shown in Figure 4 are highlighted in yellow.

**Table S5. List of primary and secondary antibodies used for western blot**.

**Table S6. List of primers and taqman probe used.**
